# Inferring test models from user bug reports using multi-objective search

**DOI:** 10.1007/s10664-023-10333-8

**Published:** 2023-06-20

**Authors:** Giovani Guizzo, Francesco Califano, Federica Sarro, Filomena Ferrucci, Mark Harman

**Affiliations:** 1 University College London, London, UK; 2 University of Salerno, Salerno, Italy

**Keywords:** Model inference, Search-based software engineering, Multi-objective optimisation

## Abstract

Bug reports are used by software testers to identify abnormal software behaviour. In this paper, we propose a multi-objective evolutionary approach to automatically generate finite state machines (FSMs) based on bug reports written in natural language, to automatically capture incorrect software behaviour. These FSMs can then be used by testers to both exercise the reported bugs and create tests that can potentially reveal new bugs. The FSM generation is guided by a Multi-Objective Evolutionary Algorithm (MOEA) that simultaneously minimises three objectives: size of the models, number of unrealistic states (over-generalisation), and number of states not covered by the models (under-generalisation). We assess the feasibility of our approach for 10 real-world software programs by exploiting three different MOEAs (NSGA-II, NSGA-III and MOEA/D) and benchmarking them with the baseline tool KLFA. Our results show that KLFA is not practical to be used with real-world software, because it generates models that over generalise software behaviour. Among the three MOEAs, NSGA-II obtained significantly better results than the other two for all 10 programs, detecting a greater number of bugs for 90% of the programs. We also studied the differences in quality and model performance when MOEAs are guided by only two objectives rather than three during the evolution. We found that the use of under-approximation (or over-approximation) and size as objectives generates infeasible solutions. On the other hand, using as objectives over-approximation and under-approximation generates feasible solutions yet still worse than those obtained using all three objectives for 100% of the cases. The size objective acts as a diversity factor. As a consequence, an algorithm guided by all three objectives avoids local optima, controls the size of the models, and makes the results more diverse and closer to the optimal Pareto set.

## Introduction

Natural language bug reports are important resources for testers to generate test cases that exploit the buggy features. Usually, once a bug report is submitted, the developers have to analyze and validate it, and later fix the bug by coding a software patch. However, it remains challenging to software testers to generate proper test cases to exercise the bugs reported by the users. As a consequence, the patches may be incomplete or even introduce more bugs in the software (Yin et al. 2011). Creating proper tests that exploit the buggy features is crucial to improve the quality of bug fixes.

In order to reduce the human effort, bug reports can be used as events to automatically generate a behavioural model of a system (Zhang et al. 2015). Behavioural models create an abstraction of a system which describes its internal behaviour. This abstraction is used to synthesize concrete software development (e.g., test cases) in an automated fashion (Boussaïd et al. 2017). The focus of these models is on the dynamic view of the system, not on how it is implemented (Dennis et al. 2015). More specifically, state machines are good behavioural models for capturing state changes of a system (Dennis et al. 2015).

Models are highly used in software engineering because they help gain a better understanding of the system for which they are built, thus helping to achieve better quality and improve productivity throughout the software development cycle (Boussaïd et al. 2017). Furthermore, by using models, developers are not forced to understand the system in terms of its source code to perform verification and validation. A behavioural reference model that contains many bug traces of an application could help developers to fix reported bugs as well as exercise unforeseen behaviour and potentially discover new bugs. We herein refer to behavioural models that aid the tester during the testing task simply as “test models”.

The automatic generation of such a behavioural model is defined as model inference. It has been introduced to reduce or eliminate the human effort involved in creating useful models, which is the major issue in terms of cost and time effort (Walkinshaw et al. 2013a). The inference technique’s performance depends on factors such as the “richness” of the input, or the complexity of the software system. Generally speaking, the generation of a model is based on two main parts: i) the selection of “examples” of software behaviour (e.g., program traces) as input data; and ii) the application of algorithms from the domain of Artificial Intelligence (Walkinshaw et al. 2013a).

Even though inference techniques are capable of automatically generating a set of solutions in an effortless way for humans, the resulting models inferred from logs that guide the testers in test cases generation can suffer from two main issues: under-approximation and over-approximation (Tonella et al. 2012). The former means that the generated model is not able to recognise some or all of the traces given for the training (under generalising). The latter means that the model is over generalising the application behaviour, i.e., generating execution paths which are not observable in practice. An example is the tool KLFA (Mariani and Pastore 2008). This tool is able to create a behavioural model of a system by using the software’s log files. However, the incremental approach for the model creation generates a model that is not practical for testers to use because of its huge size and over approximation.

This can be improved by adopting a different technique of model inference which can lead to more feasible and practical models. In this paper we explore the use of Multi-Objective Evolutionary Algorithms (MOEAs) to infer models considering multiple objectives, thus generating optimal models with a good trade-off between such objectives. Our proposed MOEA approach takes events (i.e., execution logs extracted from bug reports) written in natural language as inputs and generates finite state machine models, which are able to recognise the execution traces in the bug reports and generate unseen but valid behaviours of an application.^1^

Our approach aims at optimising three important characteristics of the generated model: their size, under- and over-approximation. The aim is to generate models that generalise the set of user events which triggered software bugs. Hence, the tester can automatically infer a behaviour model from bug reports that can guide their testing activity.

More specifically, two major use cases are possible with such models: 1) after the user reports a bug, the tester evaluates it against the inferred model; if the model recognizes it, the tester can generate the test case for that bug and eventually develop a patch; and 2) the tester examines all the execution traces generated by the inferred model in order to create new test cases to potentially find new bugs.

In other words, the idea is that the generated models can be analysed by the tester for them to better understand the steps the software takes when a bug is present. If the tester observes a state transition in the model that should not happen, they can create test cases to capture this behaviour and check whether it produces an error. If a trace derived from a bug report is accepted by the model, meaning that it is recognised by the model and observed in practice, then the tester has an indication that it is a known bug or part of a known bug. In this sense, the tester can avoid re-testing known bugs that may be already under fixing efforts, or can use the model to leverage on information of known bugs to check whether they have resurged in later versions of the software. Ultimately, these models could also be used by a test generation tool to automatically generate test inputs that are likely to capture bugs.

The main novelty of this approach lies in its generation of models based on bug reports (as opposed to execution logs as done by KLFA). This feature focuses on actual bug reproduction steps, and consequently generates models that are specific for testing. Moreover, because our approach also includes a size objective (minimisation) during the generation process, the models become smaller and produce fewer execution traces. As a consequence, the engineer can focus on specific traces that are more likely to trigger bugs, thus making the generated models more practical.

We implement three different MOEAs with our approach, namely Non-dominated Sorting Genetic Algorithm II (NSGA-II) (Deb et al. 2002), Non-dominated Sorting Genetic Algorithm III (NSGA-III) (Deb and Jain 2014), and Multi-Objective Evolutionary Algorithm Based on Decomposition (MOEA/D) (Zhang and Li 2007). Later we compare it with the baseline tool KLFA on 10 real-world programs retrieved from the BugZilla reporting system. The solutions generated by the different MOEAs are compared in terms of Hypervolume (HV) and Inverted Generational Distance (IGD) as performance indicators (Riquelme et al. 2015). We also compare them in terms of bugs revealed and overall estimated human effort on analysing the model traces to create new test cases. The main goal is to evaluate the trade-off between under and over generalisation of automatically generated models, while also assessing the fault revealing capability of each algorithm.

As studied in a previous work by Sarro et al. (2016), it is also interesting to study the benefits gained from a multi-objective formulation. Therefore, we investigate the use of different combinations of objectives: i) under-approximation, over-approximation, and size; ii) under-approximation and size; iii) over-approximation and size; and iv) under-approximation and over-approximation. With this analysis, we intend to assess whether the three objectives are indeed conflicting, or if a simpler two objective formulation subsumes the three objectives formulation.

The results show that NSGA-II, compared with NSGA-III and MOEA/D, has lower size values in 70% of the datasets, lower number of edges in all training sets of data, lower over-approximation in six out of ten datasets, and lower under-approximation in 80% of the datasets. Therefore, NSGA-II has a lower model complexity in terms of size, it produces solutions which do not over-generalise as often and, lastly, the solutions are less under-generalising, meaning that more application’s behaviours are recognized. In a real-world testing scenario, NSGA-II can reveal a higher total number of bugs than NSGA-III and MOEA/D on 80% of the testing datasets, thus producing solutions which are more extensible to new behaviours and not too specific for an application.

Furthermore, the results of the comparison between different objectives formulations show that the three objectives formulation generates smaller and more diverse solutions which can find more bugs (double the number on 70% of the datasets). The three objectives formulation yields significantly large and better HV and IGD values than the two-objectives one for all programs. We also found out that the size objective in a three objectives formulation is necessary in order to control the number of states of the automata, while also improving the solutions diversity and uniqueness. Consequently, such formulation avoids local optima during the evolutionary process and leads to more powerful models.

The main contributions of this paper are:

1. the proposal of a novel Multi-Objective Model Inference approach;
2. a large scale empirical study with real-world programs to assess the feasibility and effectiveness of our approach;
3. the comparison of three evolutionary algorithms NSGA-II, NSGA-III and MOEA/D to establish which one is the most effective on the presented model inference multi-objective optimization problem;
4. the evaluation of the benefits gained from a three-objective problem formulation compared to a two-objective formulation. The evaluation is performed in terms of solution trade-offs between under-approximation and over-approximation along with fault revealing capability of the models;
5. the comparison with a baseline tool for model inference: KLFA (Mariani and Pastore 2008);
6. the provision of our approach as an open-source software at https://github.com/SOLAR-group/ModelInference.

The rest of the paper is organized as follows. Section 2 presents the background. Section 3 shows how the proposed approach works. Section 4 presents the experimental set up. Section 5 shows the results and answers the research questions. Section 7 describes related work in the field of model-based testing using Natural Language Processing (NLP). Finally, Section 8 draws conclusions and outlines future work.

## Background

In this section we present the necessary background on Model Inference and Multi-Objective Evolutionary computation for a better understanding of the rest of the paper.

### Model inference

Model inference is a crucial part of Model-Based Testing (MBT), which aims at manually or (semi-)automatically producing (and sometimes also executing) test cases for a given system under test (SUT), based on models derived from the systems. To these end, many different approaches have been proposed in the literature. Among these, there are search-based optimization algorithms (Boussaïd et al. 2017) such as Genetic Algorithms (GAs), Ant Colony Optimization (ACO), and Simulated Annealing (SA).

There are different types of models that can be exploited to derive test cases, ranging from Unified Modelling Language (UML) (Ahmad et al. 2019) diagrams to Finite State Machine (FSM) (Paradkar 2004; Stobie 2005). Recent studies have shown how models can be also be built from artifacts written in natural language, which requires a preprocessing step to convert natural language artifacts into structured data to give as input to the model. Testers benefit from NLP techniques because the manual effort of extracting test cases from natural language requirements is reduced. Garousi et al. (2020) performed a systematic literature mapping of NLP techniques exploited in the area of NLP-assisted software testing. Substantial effort has been put on MBT, especially in devising algorithms for generating system models such as Deterministic Finite Automaton (DFA) (Dias Neto et al. 2007).

A DFA model is an automaton that mimics a given behaviour, in our case, a software behaviour. Formally (Rivest and Schapire 1993), a DFA model *M* is represented by a 5-tuple:

1 $$\begin{aligned} M = (Q, \Sigma , \delta , q_0, F) \end{aligned}$$

where *Q* represents a finite set of states, $\Sigma$ a finite alphabet, $\delta$ a transition function $\delta = Q \times \Sigma \rightarrow Q$, an initial state $q_0 \in Q$, and a set of accept states $F \subseteq Q$. In summary, a DFA model *M* receives as input a string $w = \sigma _1, \sigma _2, ..., \sigma _n$ where $\sigma \in \Sigma$, consumes each $\sigma \in w$ in order, and uses such character as input to transition from one state $q \in Q$ to another according to the transition function $\delta$ until there are no more characters left in *w*. The transition function maps each combination of $(q, \sigma )$ into a resulting state *q*, i.e., determines what is the next state $q_{i+1}$ from the current state $q_i$ when using the next input $\sigma _{i+1}$. After processing the string *w*, a final state $q_f$ is reached and, if $q_f \in F$ (i.e., the final state is an accept state), then the model *M* is said to *accept* *w*. If $q_f \not \in F$, then *M* is said to *reject* *w*. In this work, we also deal with incomplete DFA models during their inference. A DFA model is called incomplete if the transition function $\delta$ is not able to map any combination $(q, \sigma )$, i.e., it means that the model does not support the given transition. If such transition is required in practice, the model will halt and thus will not accept the string *w*.

A model inference technique must infer the elements of the 5-tuple model *M* using a set of input examples *W*. According to Rivest and Schapire (1993), the objective of a model learner is to infer a *perfect* model *M*, i.e., the inferred model can perfectly predict the correct output for any sequence of inputs $w \in W$. For that, the technique must first ensure that the inferred models comply with the rules of a DFA: i) there must be an initial state $q_0$; ii) $\delta$ is able to map all combinations of (*q*, *a*); and iii) $F \ne \emptyset$. Besides being able to infer a perfect model, the technique has to also be evaluated in terms of speed of model inference and size of learned automata (Meinke and Walkinshaw 2012), otherwise the technique itself becomes infeasible. Finally, a trade-off has to be made between the computational complexity of model inference and the cost of the models, otherwise the engineer might not be able to understand and use such inferred model for their needs. In order to balance the cost of this procedure and the quality of the results, the use of metaheuristics has been proposed (Zhang et al. 2015; Tonella et al. 2012).

### Multi-Objective Evolutionary Algorithms

Evolutionary computation comprises different kinds of algorithms such as: Genetic Algorithms (GAs), Evolutionary Programming (EP), Evolution Strategies (EAs), and Genetic Programming (GP). These algorithms address abstract tasks, representing them as search problems in a space of potential solutions. What we look for is “the best” solution, so the problem can be seen as an optimization process (Dasgupta and Michalewicz 1997).

When the search space is not feasible for classical exhaustive techniques, the need of more sophisticated artificial intelligence techniques such as Evolutionary Algorithms arises. They are naturally stochastic and the search methods derive from natural phenomena like genetic inheritance and Darwinian strife for survival (Dasgupta and Michalewicz 1997).

The behaviour of an EA comprises of a population which evolves over several generations, following the natural selection and reproduction. Each individual in the population represents a solution, which is differentiated from the others by its chromosome. A chromosome, constituted by a set of genes, can be represented as an array of simple data type like bit, float or integer, or a complex data type like objects. The population evolves over several generations, and two essential operations are stochastically applied: crossover and mutation. The crossover operator takes a set of parents and generates new individuals which carry the genes of their parents. The mutation operator promotes diversity, applying a perturbation to the genes of the individuals. At each generation, the solutions are evaluated using a fitness function, which denotes the quality of the solution. The process is repeated until a stopping condition is met, which can be a specific fitness value, running time, or number of evaluations.

EAs are based on an iterative optimization process which produces a certain number of solutions in the search space. In our case, a solution is represented by a model which, starting from artifacts written in natural language, can be inferred by adopting an optimization technique.

In an optimization problem, a solution is described in terms of a decision vector $(x_1, ..., x_n)$ in the decision space $X$ (Zitzler et al. 2004). Each solution’s fitness is represented by an objective vector $(z_1, ..., z_k)$ in the objective space $Z$, assigned by a function $f: X \rightarrow Z$. Optimization problems aim to minimize or maximize one or more objectives, depending on the particular problem. An optimization technique can be mono-objective or multi-objective.

The former type is used when the individuals are evaluated on just one value that denotes their fitness. The optimization process is simpler because the solutions are ranked with respect to their fitness, and the best ones are selected for the mating operations and for survival.

On the other hand, when dealing with a multi-objective problem, the optimization technique generates a set of diverse solutions. Since there is not a single best solution, the concept of Pareto dominance is used. For minimization, a solution $x_1$ is said to dominate a solution $x_2$ $(x_1 \succ x_2)$ if no objective $z_i$ of $x_1$ is greater than $z_i$ of $x_2$ and at least one objective is smaller (Zitzler et al. 2004). If these conditions do not hold, the solutions are called “non-dominated”, meaning that both are equally acceptable solutions with different trade-offs for the problem.

Hence, the result of a multi-objective optimization is a set of optimal solutions. The set of optimal solutions in the decision space $X$ is in general denoted as the *Pareto set* $X* \subseteq X$ and its image in the objective space is denoted as *Pareto front* $Z* = f(X*) \subseteq Z$. The Pareto set has to be analysed by the decision maker in order to find the solution with the best trade-off between objectives values for the particular problem, based on their needs. The aim of evolutionary multi-objective optimization is to find a good approximation of the Pareto set, because often the real Pareto front cannot be found in feasible time.

A very popular MOEA is NSGA-II, which performs the selection and survival operations using the concept of crowding distance (Deb et al. 2002). For the replacement operation, the individuals ranked first based on the dominance relation and then based on their crowding distance. The NSGA-III algorithm uses reference directions to guide the search (Deb and Jain 2014). NSGA-III introduces significant changes to the NSGA-II algorithm, maintaining the diversity of the population members by supplying and adaptively updating a number of reference points. A very different approach is used by the MOEA/D algorithm which is based on decomposition (Zhang and Li 2007). The multi-objective optimization problem is decomposed into a number of scalar sub-problems, which are optimized simultaneously by the algorithm. The sub problems are optimized using information about their neighbours sub problems.

In this work, we use these three MOEAs to generate testing models (according to the representation shown in Equation 1) from bug reports written in natural language. We chose these MOEAs as they are well-known in the literature, and have been used in the past for various Search-Based Software Engineering (SBSE) problems including software testing (Tonella et al. 2012; Guizzo et al. 2020), software effort estimation (Sarro et al. 2016; Tawosi et al. 2021) and software project management (Ferrucci et al. 2013; Sarro et al. 2017).

## Proposed approach

**Fig. 1:**
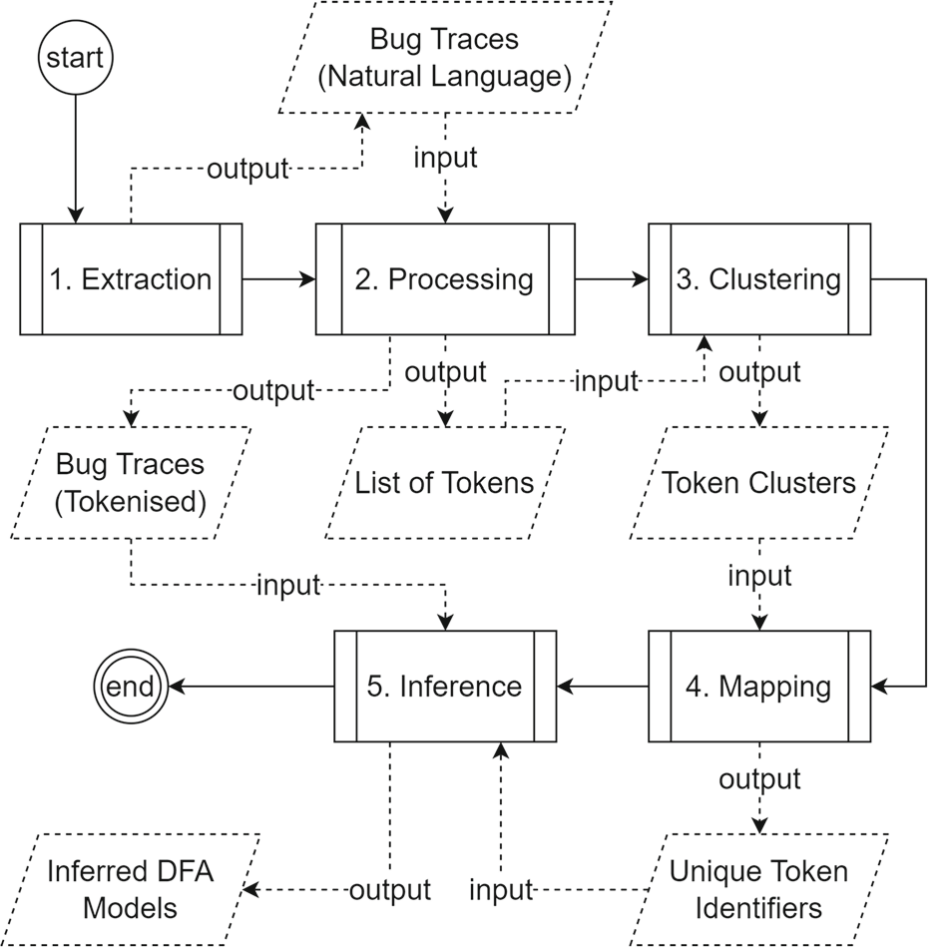
Approach overview. Solid boxes represent processes and solid lines transitions between them. Dashed boxes represent artefacts and dashed lines represent inputs and outputs of given artefacts

The main goal of this work is to provide an approach that performs all necessary tasks to achieve a behavioural model for a SUT without any additional information other than the bug reports themselves (written in natural language). Such behavioural models can help engineers in creating test cases that can help them exercise defective code or reveal new faults. As far as we are aware, only Zhang et al. (2015) were able to provide a prototype tool that could aim at such a goal. Our approach is built upon the initial efforts of Zhang et al. (2015); it consists of five main phases, as shown in Fig. 1 and briefly explained next and thoroughly explained in their subsequent subsections.

The first step of our approach is named **Extraction** (Section 3.1). Given a bug tracking system URL, it crawls the system to retrieve a list of bug reports written in natural language. It then filters the reports to retain only the reproduction/trace steps representing the behaviour we want to model. As output, it generates a list of bug traces which is given as input to the next phase. In this paper, we consider “trace step” as a single step of a given bug report, and “bug trace” the full list of steps to reproduce the bug.

The second phase is called **Processing** (Section 3.2). The main goal of this phase is to parse the bug traces written in natural languages with an NLP framework in order to create a more structured set of bug traces. This is required to allow for a more effective model inference procedure. The result is a list of tokenised bug traces/reports and a list of known tokens. The latter is given as input to the next phase.

The third phase, **Clustering** (Section 3.3), aims at clustering similar trace steps in order to identify the same behaviour. Hence, all trace steps inside a cluster represent the same software action. This cluster of tokens is given as input to the next phase.

The fourth phase of our approach is named **Mapping** (Section 3.4). This phase maps clustered trace steps into a single step, i.e., it assigns a single unique identifier to all steps of a given cluster, consequently reducing the number of redundant steps and simplifying the models.

Finally, the fifth phase, **Inference** (Section 3.5), is where the inference of the DFA models is done using MOEAs on the trace steps obtained in the previous phases. It receives as input the list of tokenised bug reports/traces (generated in phase two) and the list of unique tokens identifiers (generated in phase four). The latter represents the alphabet/input space $\Sigma$ of our DFA (see Sections 2.1 and 3.5.1). The former is what our algorithm aims at modelling with the inferred models, i.e., the goal is to create models that can mimic the behaviours represented by such reports. These generated models are given as output to the engineer, who can use such models to create test cases that can exercise the faulty code or generate unseen traces to reveal new bugs.

### Extraction

In the first phase, we extract bug reproduction information from the bug tracking system. For each SUT, we access its bug tracking system and execute a *webcrawler* to mine the existing bug information. Each bug report is transformed into a raw HTML page.

The relevant information obtained in this phase are the steps reported to reproduce the bug. These steps are usually located between the “Steps to Reproduce”and “Actual Results” (or sometimes “Expected Results”) keywords. We have analysed the bug tracking systems used in this work and found out that, although not officially disclaimed, there seems to be a convention by bug reporters and developers on how to report bugs this way. We used this convention to obtain a more accurate bug extraction. Hence, we filter the raw HTML pages to contain only this information and then transform such pages into a JSON file.

**Fig. 2:**
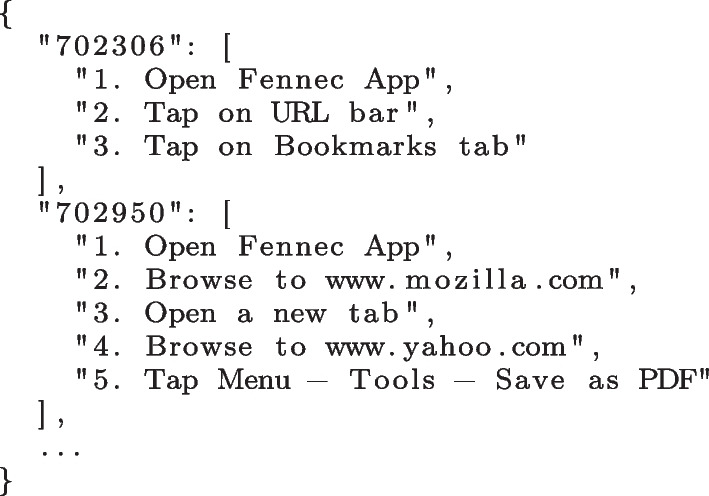
Raw bug reproduction steps

Each JSON file represents a SUT containing a list of bugs. A bug is identified by its ID (key) and is associated to a list of steps to reproduce (value). Figure 2 depicts an example of such file for the “Firefox for Android” program (see Section 4 for more information on the datasets used in this work).

### Processing

The second phase focuses on processing the data collected during the bug report extraction in order to remove noise and create tokenised traces. This phase is needed to retain only steps that can translate into actionable operations in the software.

We first remove noise by deleting the leading step numbers, sentences in parenthesis (which usually contain complementary information), and splitting composite trace steps into multiple steps (e.g., step 5 of bug 702950 in Fig. 2). Next, we transform each step into a list of words by tokenising the sentences. For that purpose, we use the NLTK framework.^2^ We then remove stop words (e.g., “is”, “the”, “at”) and punctuations. Finally, we stem the words to their roots. Stemming is a common NLP technique to reduce multiple similar words into a single stem for better recognition. For instance, the words “browse”, “browsing”, and “browsed” are all stemmed to “brows”.

Moreover, we also remove words that appear only once in the dataset, because if they were included, we would have an overly long dataset and traces containing steps that do not represent a step shared by many bug reports. In this sense, such words may be part of specific steps that serve as complement of a previous step or by incorrect steps wrongfully observed by a single user, and thus they are likely to be irrelevant.

**Fig. 3:**
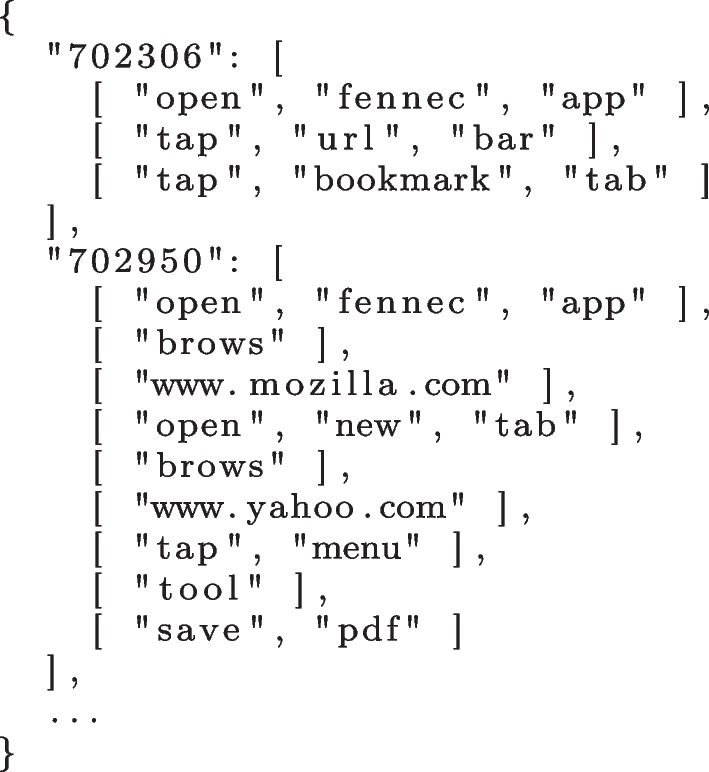
Processed bug reproduction traces

At the end of this step, we obtain a list of traces comprised of lists of words. Figure 3 depicts the results of the data processing procedure, when applied to the reproduction steps presented in Fig. 2.

### Clustering

It is very unlikely that all users will use the same words to describe the same software behaviour, hence we must perform a clustering of similar words to identify synonyms. With this in mind, we first perform a Latent Semantic Analysis (LSA) (Dumais 2004). Briefly, LSA creates a matrix of words and documents (i.e., steps), where each cell is the frequency of the given word in a given document.

The clustering algorithm uses the information retrieved by the LSA procedure and projects it into a Latent Semantic Indexing (LSI). This indexing is then used by the algorithm to compute the similarity between the steps and then join them into clusters. Since the number of clusters *k* is defined by the user, we had to set it as an additional parameter. In a preliminary experiment, we found out that the best value for *k* is around 20%–35% of the total number of steps in the dataset, to a maximum of 400 clusters. A second parameter is the cosine similarity threshold which was set to 0.7, i.e., the threshold to consider two trace steps similar. These values come from an earlier preliminary experiment and from what we observed in related work (Zhang et al. 2015).

**Fig. 4:**
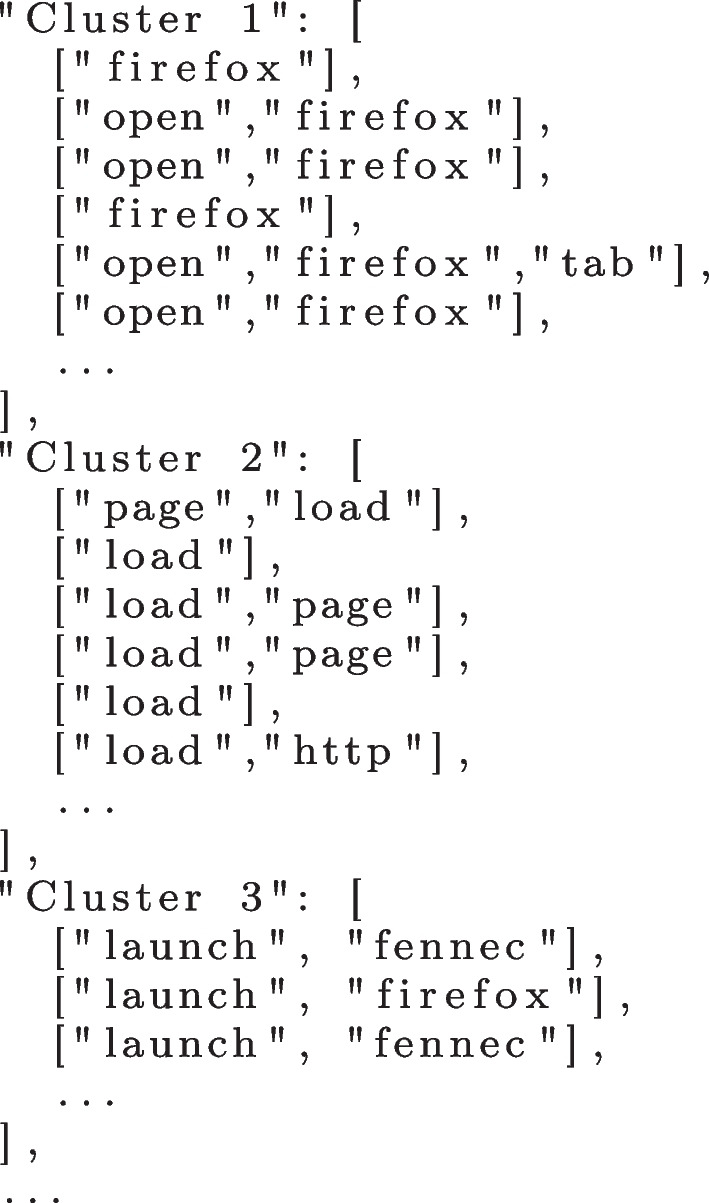
Clusters of similar trace steps

The clustering algorithm iteratively performs LSI and compares the similarity of the trace steps in a pairwise fashion. When a pair of steps is deemed similar (i.e., meets the threshold 0.7), then they are added to the same cluster. After comparing all pairs, the algorithm compares the entire clusters of steps generated in the previous iteration. When it finds two clusters of trace steps that are sufficiently similar (0.7 threshold), they are joined into a single cluster. Repeated steps are removed and overlapping clusters have their intersecting items re-clustered. This process continues until there are no more changes in the clusters. Figure 4 depicts an example of clusters of trace steps obtained from the data shown in Fig. 3.

### Mapping

After obtaining a converged set of clusters, the next step is to map each cluster into a distinct and unified step identifier that can represent all trace steps of such cluster. With this we can then represent similar trace steps that are worded differently into a single software behaviour during the model inference. The adopted mapping follows a simple heuristic: i) if all steps in a given cluster are equal, use the trace step as identifier; else ii) if all trace steps are of length one (i.e., only contain one word), use the most frequent one as identifier; else iii) compute the most frequent trace step length and use the most frequent collocation of steps (up to quadgram collocation) as identifier.

In the example shown in Fig. 4, “Cluster 1” is mapped to *[“open”, “firefox”]* (or in a single word *“open_firefox”*) because the most frequent trace step size is two and this is the most frequent bigram collocation of words. Similarly, “Cluster 2” is mapped to *[“load”, “page”]* and “Cluster 3” is mapped to *[“launch”, “fennec”]*.

**Fig. 5:**
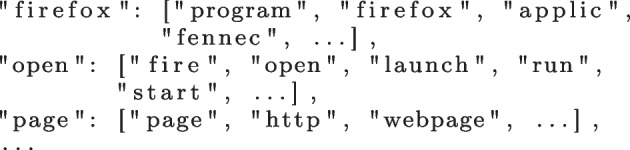
Dictionary of synonyms for Firefox for Android

However, this mapping can miss clusters with the same meaning or even with typos, which we have indeed observed. For example, the word “fennec” is the codename of the Firefox app for Android, which is a specific jargon for that program. A typo such as “fenec” is also possible. Hence, the identifier *[“open”, “firefox”]* and *[“launch”, “fennec”]* refer to the same step/action of opening the browser, but they are in different clusters. In this situation, the algorithm generated two distinct clusters with trace steps representing the same behaviour, but with different words identifying them. To solve this problem, we defined a dictionary of synonyms for each program and included those corner cases, as shown in Fig. 5. The creation of this dictionary is the only part of our approach that is not automated and can incur some manual effort depending on the size of the dataset. Future work can consider automating the creation of such a dictionary.

**Fig. 6:**
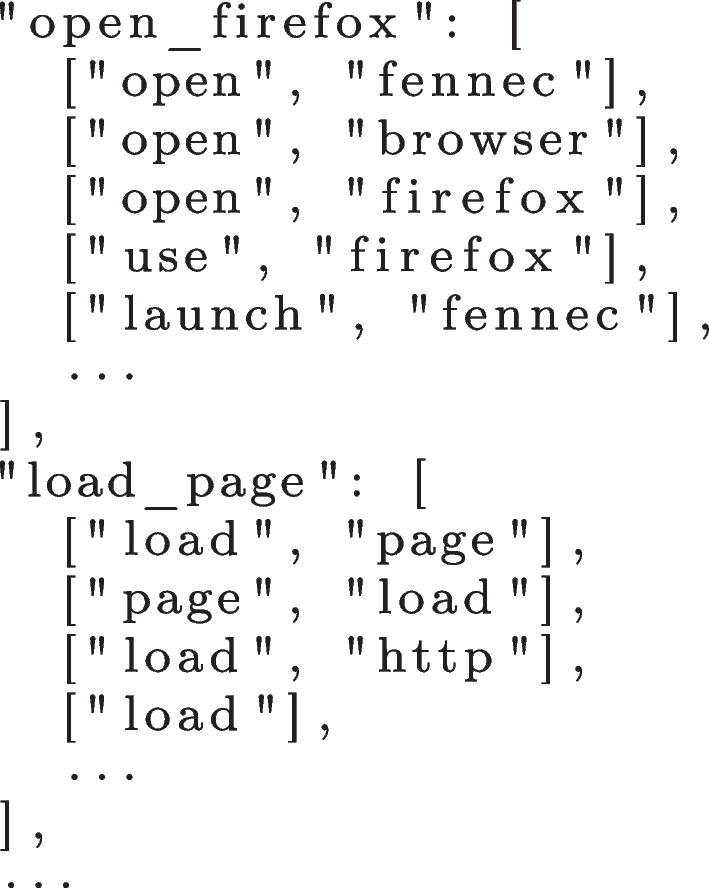
Mapped clusters of trace steps

Then, using the dictionary, we unified clusters with the same meaning. At the end of this procedure, we obtained a set of clusters of unique trace steps representing the unique steps to reproduce the bugs. Figure 6 depicts an example of the final format of the clusters.

Using these clusters in conjunction with the processed bug reports data, we are able to identify which bug reproduction steps translate to which distinct trace steps. This data is then given as input to the next phase of our approach.

### Inference

In the final phase of our approach, we execute MOEAs (Section 2.2) in order to generate optimised DFA models that can represent the behaviour of the SUT. In other words, we want to generate DFA models that can represent the behaviour described by the bug reports, but at the same time we want those models to be small in size, whilst also under- and over-generalising as little as possible. This is a naturally multi-objective problem, since these objectives are typically conflicting (Harman 2007).

DFA models suit our context because they can succinctly represent the steps of a bug report: it has states connected to each other representing current software states, and the transitions represent actions/trace steps taken by the user, there is an end to the reproduction steps, and nodes can be connected to many other nodes due to the possibility of many steps creating the same state. Moreover, since they can be represented as graphs, it allows us to apply optimisation techniques more easily.

Next we describe the main components of our algorithm and how to use it to automatically infer models.

#### Solution representation

One of the most important components of a MOEA is the solution representation (Konak et al. 2006). As we mentioned before, each state represents a software state and the transitions between states are actions taken by the user (as described in the bug reports). Hence, a DFA is capable of emulating the software behaviour behind a bug report by incorporating the set of bug report steps (trace steps) as automaton transitions and the consequent erroneous software state as the final DFA state (accepting node).

In our case, our DFA model is represented slightly different from the conventional 5-tuple model (Equation 1). Recapitulating, the original DFA model can be represented as $M = (Q, \Sigma , \delta , q_0, F)$ and has to adhere to three rules: i) it must have a start state $q_0$; ii) the transition function $\delta$ must be able to map all combinations of states and inputs $Q \times \Sigma$ to a state in *Q*; iii) and the set of final states *F* must not be empty. Our modified DFA representation, which is an adaptation from the works of Tonella et al. (2012) and Zhang et al. (2015), includes a few constraints that make the DFA model more consistent with a software behaviour:

2 $$\begin{aligned} M' = (Q, \Sigma , \delta ', q_0, q_f) \end{aligned}$$

where *Q* is the set of all software states, $\Sigma$ is the set of all known trace steps (created according to our mining approach presented in the previous sections), $\delta '$ is the modified transition function $\delta ' = Q \times \Sigma \rightarrow Q \vee \emptyset$, $q_0 \in Q$ is the starting state, and $q_f \in Q$ is the end/accept state.

First, instead of a set of accept/final states *F*, our DFA model $M'$ contains only one final state $q_f$, which represents an erroneous state in the SUT. If a given execution trace is fed into the model and the execution does not ent at $q_f$ (rejects the trace), then it means that the software behaviour has not been observed in practice or it likely does not result in an erroneous state. It is worth noting that the final state $q_f$ can also act as an intermediate state of a given trace.

Another difference is the modified transition function $\delta ' = Q \times \Sigma \rightarrow Q \vee \emptyset$. Differently from the original function $\delta$, the modified version $\delta '$ may yield an empty result when mapping a combination $(q, \sigma )$ of state and trace step/input. In other words, it is possible that from a given state *q*, the trace step $\sigma$ cannot be executed, which constitutes an unobserved or impossible behaviour. Using the example of Firefox given in the previous sections, an example of unobserved (and likely impossible) behaviour would be $(q_0, load\_page )$, i.e., one cannot start the trace execution by trying to load a page before even opening the program (*open_firefox*). In this case, the DFA model halts and rejects the execution trace. This modified function of our representation violates the aforementioned second rule of a DFA, i.e., our representation allows for the creation of incomplete DFAs during the inference procedure. In fact, the perfect model $M'$ will likely be incomplete, since SUTs most likely contain constraints that prevent the execution of specific trace steps from specific states (much like the Firefox example). Complete models can also be generated with our approach, however they are less frequent than incomplete models because our approach allows for the direct generation of incomplete models as opposed to generating a complete model and then pruning it.

**Fig. 7:**
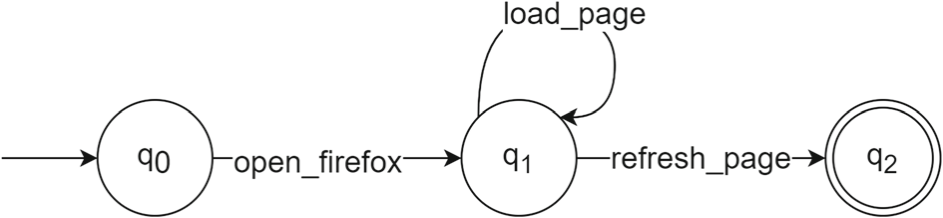
Example of a DFA model

Figure 7 depicts a simple example of a DFA model $M'$ in our context for the Firefox application. In this hypothetical scenario, the refresh feature of the program is defective, and thus anyone who tries to perform the action *refresh_page* will encounter a failure and the software execution will halt.

The model contains the following nodes $Q = \{q_0, q_1, q_2\}$, where $q_0$ is the initial state, $q_1$ is an intermediate state, and $q_2$ is the final state $q_f$. The possible transitions (trace steps) are $\Sigma =$ {*open_firefox*, *load_page*, *refresh_page*}. The transition function $\delta '$ of the example model $M'$ can perform the mappings shown in Table 1.

**Table 1:** Transition function $\delta '$ for the example of Fig. 7. Transitions $\sigma \in \Sigma$ are presented in rows, states $q \in Q$ in columns, and each cell contains the result of the state-transition (*q*, $\sigma$) mapping, i.e., the resulting state

	States		
Transitions	$q_0$	$q_1$	$q_2$
open_firefox	$q_1$		
load_page		$q_1$	
refresh_page		$q_2$	

In this example, the first encountered constraint is that the user must perform the *open_firefox* step before any other, hence, this is the only possible transition from $q_0$. By doing so, the execution moves to the intermediate state $q_1$. From $q_1$, there are two possible transitions: i) $(q_1, load\_page ) \rightarrow q_1$; and ii) $(q_1, refresh\_page ) \rightarrow q_2$. The final state $q_f = q_2$ can only be reached once the user performs the defective action *refresh_page*, after which the erroneous state is reached and the program ends. However, the example model allows for the execution of the *load_page* as many times as required.

In this scenario, trace inputs such as (*open_firefox*, *refresh_page*) and (*open_firefox*, *load_page*, *refresh_page*) are accepted by the model, but traces such as (*open_firefox*, *refresh_page*, *refresh_page* — inexistent transition in $\delta '$) and (*open_firefox*, *load_firefox* — ends in a node different than $q_f$) are not accepted. Moreover, if one uses the model to generate unseen traces, it will not allow the generation of traces that do not fit the 5-tuple specification.

We use the FAdo v1.3.5.1 framework^3^ in order to implement this representation. With this framework, we can easily map a graph to a finite state automaton. The MOEA will act on this representation during the evolutionary process to create candidate models as its solutions.

#### Population initialisation

The initial population of solution models is formed randomly. Each initial solution is created as union of three random bug traces with equal probability, where each bug trace is composed by a set of trace steps. First, the algorithm generates an automaton formed by three parallel chains of states and their transitions, each of which representing one of the selected bug traces, starting from the same node $q_0$, and ending on the same node $q_f$. This union procedure is the basis for the Union Crossover Operator described in the next section. Second, because such model can be a Non-deterministic Finite Automaton (NFA), we use the powerset construction algorithm (Rabin and Scott 1959) to transform it into a DFA. Then, during the evolutionary process, the algorithm recombines these initial models and mutates them (with a given probability) using crossover and mutation operators to create new generations of solutions.

#### Crossover operators

**Fig. 8:**
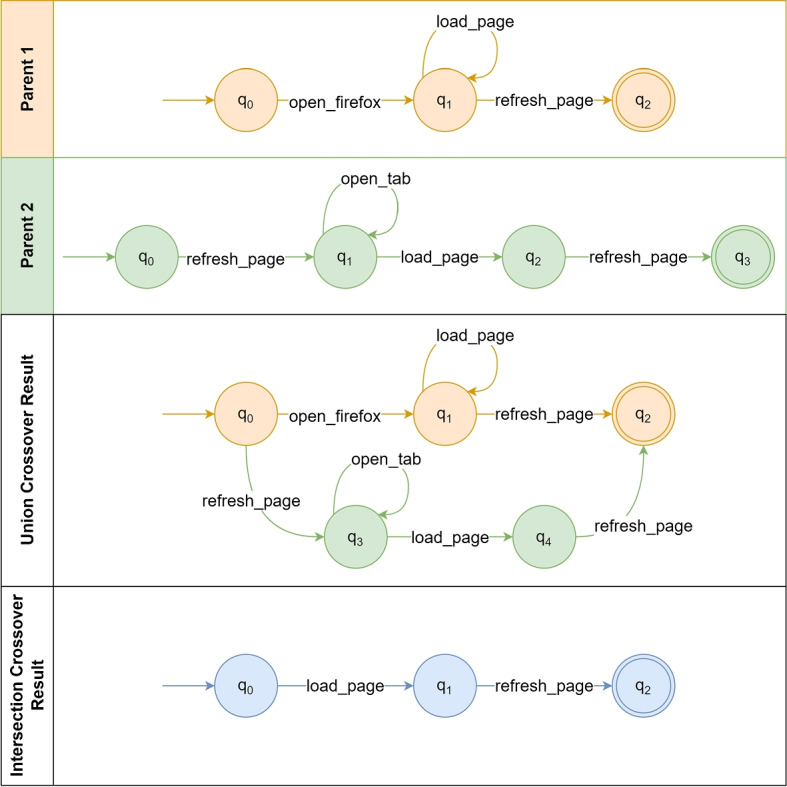
Crossover operators example. The first two horizontal lanes represent the two parents used in the crossover. The third horizontal lane depicts the result of applying the Union Crossover using Parent 1 as basis, while attaching the non-initial and non-final nodes of Parent 2. The fourth horizontal lane depicts the result of the Intersection Crossover. Intersecting nodes and transitions are highlighted in blue

We use two crossover operators which are selected at random during each reproduction step of the evolutionary algorithm: *Union* and *Intersection*. Figure 8 depicts the crossover operators used in our algorithm.

The *Union* operator combines two models by merging together their states and transitions. Given two parents, a new child solution is generated as a copy of either parent plus the non-initial and non-final states and transitions of the other parent. First, the operator copies Parent 1 into a new solution (child). Then, the second parent’s states and transitions are attached to the child’s initial state and ending on the child’s accepting state. This creates a model that can generate all the traces of the first and second parents alike. This considerably increases the size of the model, but also has the potential to reduce the number of existing bug traces that are unrecognised by such model. This operator can cause a model to become an NFA (multiple sibling transitions with the same condition). Therefore, in order to turn NFAs into DFAs, we apply the powerset construction algorithm (Rabin and Scott 1959).

The *Intersection* operator generates children containing only the intersecting transitions and nodes of both parents. In order to do that, both parents are traversed depth-first starting from their initial nodes. When a node or a transition is the same for both parents, it is copied to the child. In the example of Fig. 8, the node $q_0$ is the same for both parents, thus it is copied to the child, but the parents’ $q_0$ nodes do not share any transition. In cases like this, the algorithm copies the next matching transition to that node, which is the transition *load_page* from $q_1$. Because in Parent 1 the *load_page* transition is recursive, the Intersection operator then compares the transitions of Parent 1’s $q_1$ with the transitions of Parent 2’s $q_2$. This procedure continues until either model has no more nodes to compare. At the end, a potentially smaller child is created with lower over-generalisation of the system behaviour, i.e., only the behaviour that is common to the parents is passed along the generation.

#### Mutation operators

**Fig. 9:**
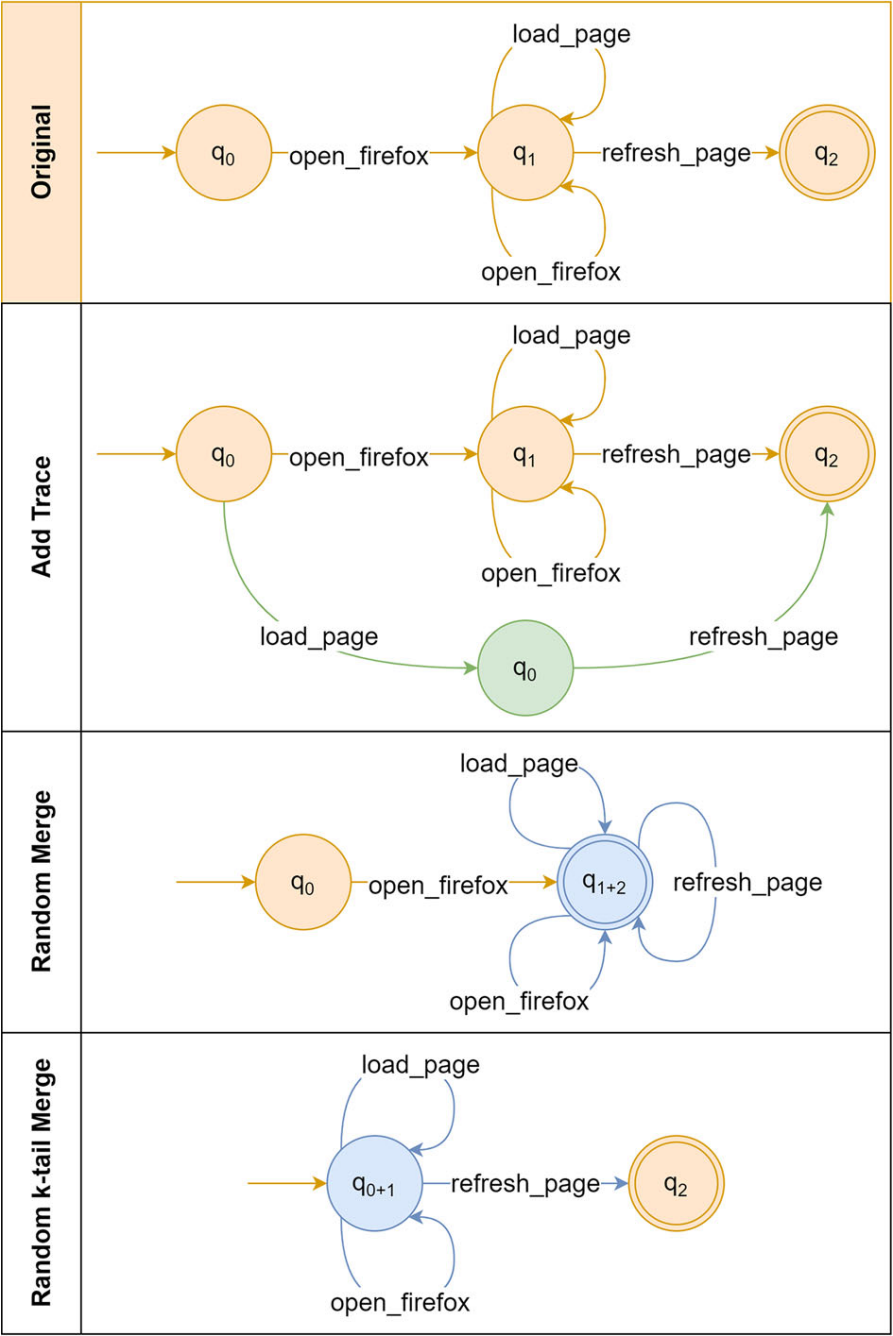
Add Trace and Merge State operators examples. The first horizontal lane represents the model before any mutation. The second and third horizontal lanes represent the model after the mutations using the Add Trace and Merge State operators, respectively. Merged nodes and transitions are highlighted in blue. Newly created nodes and transitions are highlighted in green

For the mutation procedure, we apply two different operators chosen at random with the same probability: *Add Trace* and *Merge State*. Figure 9 depicts examples for the mutation operators used in our algorithm.

The *Add Trace* operator modifies a given model in order to make it accept a random bug report/trace. Using the example of Fig. 9, let us assume the trace *load_page* $\rightarrow$ *refresh_page* was randomly selected. Because the original model would not accept such a trace (there is no transition *load_page* from the initial node), then the operator performs the mutation to force the model to accept the selected trace. It works by first creating a simple linear model for the selected trace, and then using the Union Crossover operator to merge both of them. The result of the example is similar to the Union Crossover example of Fig. 8, as both use the same model merging procedure. This mutation can potentially improve the under-generalisation of the model, but also has the drawback of increasing its size.

The *Merge State* is composed of two sub-operators, namely *Random Merge* and *Random k-tail Merge* with 10% and 90% probability respectively. The former takes two nodes at random from the model under mutation and merges them into a single one with all the transitions of both nodes. In the example of Fig. 9, the nodes $q_1$ and $q_2$ are randomly selected to be merged into the new $q_{1+2}$ node. Because $q_1$ and $q_2$ are adjacent, any transition between both (*refresh_page* in the example) is transformed into a self-transition in $q_{1+2}$. Then the operator removes duplicated transitions, i.e., transitions with equal names, starting from the same state, and ending on the same state.

The *Random k-tail Merge* performs a similar merge procedure, but it only merges two randomly chosen nodes in *Q* in case the set of *k*-tails $T^k_{q}$ of one is the subset of the other. A tail $t^k_q \in T^k_{q}$ of size *k* of a given state *q* is a list such that $t^k_q = \{\sigma _0, \sigma _1, ..., \sigma _{k-1}\}$ and $\sigma _i \in \Sigma$, where each transition $\sigma _i$ is at most *i* distant from *q*. In other words, a tail of size *k* is a list of *k* transitions that can be executed starting from *q* and ending on a node at most *k* distant from *q*. We set *k* to two in our implementation. If *k* is too high, it is very unlikely that the operator will be able to find such mergeable nodes. In the example of Fig. 9, the set of 2-tails of $q_0$ (i.e., $T_{q_0}^2$) is: i) *open_firefox* $\rightarrow$ *open_firefox*; ii) *open_firefox* $\rightarrow$ *load_page*; and iii) *open_firefox* $\rightarrow$ *refresh_page*. Because $T_{q_0}^2 \subset T_{q_1}^2$, i.e., the model allows the execution of all the sequences of transitions of $q_0$ if we start in $q_1$, then the Random k-tails Merge operator allows the merging of both nodes as done by the Random Merge operator.

The ultimate goal of Random k-tails Merge is to merge two nodes such that one is likely to subsume the other in terms of possible transitions. By doing so, the operator removes possibly redundant nodes and also introduces new transitions and nodes that allow for not only the acceptance of existing bug reports, but also the creation of unseen traces that are likely to be valid. As a consequence, this mutation is able to reduce the size of the models, to maintain existing behaviour, and to generate solutions with lower over-generalisation, as recommended in related work (Tonella et al. 2012).

After a successful mutation operator application, there is a 55% chance of an additional step: pruning. In this step, a model is minimised by using the *Hopcroft’s minimisation* algorithm (Hopcroft et al. 2007) with the purpose of removing redundant nodes and transitions. We did not try to minimise all the models with 100% probability because *Hopcroft’s minimisation* is a complex algorithm that uses a considerable amount of computational power to execute. Therefore, it can increase the execution time of our approach to a point of infeasibility for models of *Q* size 100–500 (which is commonly achieved by the algorithm). If we set the probability to 0%, we observe a faster execution, but the models grow in size in a much faster rate. All in all, the 55% minimisation rate is an acceptable compromise we found for our approach.

#### Fitness functions

In this work, we implement the three minimisation objectives: model size, under-, and over-approximation (Tonella et al. 2012). These functions measure the properties that we are trying to optimise, i.e., they measure the quality of our solutions (models). If an unrecognised/unaccepted behaviour occurs, it will lead to under-approximation, thus the model will not be able to reproduce the bug trace and the tester will not be able to properly generate tests based on the model. If the model generates unobserved behaviour, it will lead to over-approximation, thus the model will provide false positives and the tester will lose time with test cases that do not capture a real behaviour of the software. Finally, the size of the model relates to the number of traces it can generate. While generating many traces can be helpful to capture bugs, too many traces incur infeasible human effort in analysing them and creating test cases to cover such traces.

The first objective is straightforward: we want to minimise the number of states in the state machine models. It is measured by Equation 3:

3 $$\begin{aligned} \downarrow size(s) = |Q| \end{aligned}$$

where *s* is the model being evaluated, and |*Q*| measures the number of states of *s*. The smaller the model, the fewer traces it will be able to generate and the more likely it is for the tester to be able to satisfactorily interpret it.

Under-Approximation (UA) measures the number of existing bug reports not accepted by the candidate model/solution *s*. UA is computed by using Equation 4:

4 $$\begin{aligned} \downarrow UA (s, W) = \sum _{w \in W} {\left\{ \begin{array}{ll} 0 &{} { if} s\ accepts\ w \\ 1 &{} { otherwise} \end{array}\right. } \end{aligned}$$

where *W* is a set of existing bug reports/traces (possible real inputs to the model). That is, for a set of |*W*| bug reports, UA measures the number of reports $w \in W$ that cannot be accepted by *s*. The lower the UA, the less likely *s* is in rejecting existing bug reports.

On the other hand, the Over-Approximation (OA) objective measures how much behaviour is unobserved in reality. Equation 5 computes the OA of a model:

5 $$\begin{aligned} \downarrow OA (W', W) = \sum _{w' \in W'} {\left\{ \begin{array}{ll} 0 &{} { if} w' \in W \\ 1 &{} { otherwise} \end{array}\right. } \end{aligned}$$

where *W* is a set of existing bug reports/traces; and $W'$ is the set of execution traces up to size 4 that can be generated by *s*.^4^ In other words, we want to minimise the number of infeasible behaviours that can be generated by *s*. The lower this number, the more likely the traces in $W'$ are in translating into real observed behaviour.

The optimal solution would be the one that does not generate unobserved behaviours, accepts all observed traces, and is as small as possible; because such a solution is hard to find (sometimes impossible in feasible time), we expect to find solutions with a good trade-off instead. Hence, the result of one algorithm execution is a Pareto front with the non-dominated models from which the engineer can choose the solution that best fits their needs. In order to aid the engineer in choosing a solution that best suits their needs, next we provide some suggestions on interpreting the fitness values and choosing a solution.

#### Choosing a solution

Under-approximation impacts the engineer by providing them with unrecognised behaviour, i.e., behaviour that the software produces but that the model deems as incorrect. Since the model is created with bug reports and thus represents the known buggy behaviours, an unrecognised behaviour is likely to be a new bug if it is derived from a new bug report. Hence, if the tester wants to find new bugs, they should focus on analysing traces that are not accepted by the model. We minimise it during training because we want to find models as accurate as possible with the known bugs to avoid flagging a known bug as unrecognised.

Over-approximation impacts the engineer by providing them with modelled behaviours that are not real, i.e., unobserved behaviour that the model produces but are not possible in practice. Such kind of error may impact the tester by making them focus on creating test cases that do not capture real buggy behaviour and thus wasting precious testing resources. We minimise error during the model inference process to avoid the creation of irrelevant traces.

Based on these scenarios, the tester can choose a solution in the Pareto front that better suits their needs. If the tester selects a model with a high under-approximation, they will not be able to model known buggy behaviours. If the tester selects a model with a high over-approximation, they will model irrelevant behaviour. In general, the lower the fitness values, the better the model in all scenarios for known bugs.

## Experimental design

In order to assess the effectiveness of our approach, we consider the following Research Questions (RQs):

**RQ1: How good are the model inference results of MOEAs in terms of objective values and fault revealing ability when compared to the baseline tool KLFA?** This question is designed to evaluate how good the MOEAs are at inferring models which have good enough fitness values when compared to the baseline tool KLFA (Tonella et al. 2012). Moreover, by answering this question, we intend to analyse the fault revealing abilities and cost of each algorithm when compared to KLFA.

**RQ2: How do different MOEAs perform when solving this problem?** By answering this question, we intend to gain insights into the capabilities of each MOEA when dealing with this problem. Hence, we evaluate their results with multi-objective quality indicators (more details to follow in the next subsections), objective values, and their fault-revealing capabilities.

**RQ3: What are the MOEA results when formulating the problem as a two objective problem rather than a three objective one?** As detailed in Section 3.5.5, the algorithms optimise three objectives simultaneously: under-approximation (UA), over-approximation (OA), and model size. Given the different problem formulations, we want to investigate whether using 3 objectives is indeed necessary, i.e., perhaps the objectives are not entirely conflicting and a simpler formulation should suffice. Either way, by answering this question, we can better understand the problem and provide further insights that can help improve the results, consequently making the usage of this technique more practical.

The data generated and analysed during the current study are available in the UCL’s Figshare repository, https://doi.org/10.5522/04/14736180.

### Experimental subjects

In order to answer those questions, we use a set of 10 real-world programs. The datasets were collected following the first steps presented between Sections 3.1–3.4. Table 2 shows the descriptive statistics of bug’s length for each dataset/program.

**Table 2:** Program statistics for train/test set of bug reports

Program	N. bugs	Mean	Std. Dev.	Variance
Kate	401/100	5.20/5.03	3.10/2.64	9.63/6.98
Vibe	425/106	7.93/9.42	4.72/8.66	22.32/74.93
Krita	962/240	5.48/5.39	3.42/4.60	11.69/21.18
LO Writer	1,040/260	6.10/5.47	4.30/5.24	18.51/27.45
Firefox OS	1,082/271	6.00/6.18	3.55/3.94	12.63/15.53
Firefox And.	1,054/264	6.08/5.67	3.22/2.98	10.34/8.88
SeaMonkey	1,598/400	4.49/4.80	2.29/2.41	5.22/5.82
Thunderbird	1,654/414	5.22/4.99	3.32/3.00	11.05/9.02
Calendar	2,576/644	5.32/5.79	3.07/3.94	9.42/15.49
BIRT	4,361/1,090	5.85/5.68	3.20/2.94	10.23/8.64

Each dataset is divided into training and testing set for validation purposes, with a percentage equal to 80% and 20% respectively. The first slice is used for the inference process, while the second is used for the fault revealing capability analysis. Cross-validation is a common practice (Berrar 2018) when dealing with time-based data, as in our case the bug reports are retrieved in chronological order. This technique of distinguishing between a training and testing set aims to assess how the generated models will generalize to to unseen behaviours. In other words, using the same data to train and test the models would result in the unrealistic scenario where all the models would be almost completely accurate and too specific for that set of data.

### Experimental set-up

We adopt three different MOEAs in our experiments: NSGA-II, NSGA-III and MOEA/D. We chose these algorithms for several reasons. The first algorithm NSGA-II is chosen because it was adopted in previous work (Sarro et al. 2016). NSGA-III is chosen because it is an interesting evolution of NSGA-II, uses a different non-dominated sorting based on reference directions, and is deem to be better suited for multi-objectives optimisation. MOEA/D is chosen because it is a completely different evolutionary approach based on decomposition so it is interesting to compare it with the other two. Lastly, these algorithms are publicly available in the framework used for the implementation of our problem, the *pymoo* framework v0.4.2.2 (Blank 2022). This framework provides a visualization module and performance indicators like Hypervolume (HV) (Zitzler et al. 2003) and Inverted Generational Distance (IGD) (Audet et al. 2018), allowing for ease of use and replication. We also include the KLFA (Mariani and Pastore 2008) tool in our experiments as a baseline to be consistent with previous work, since the most closely related paper to ours (i.e., Tonella et al. (2012); Zhang et al. (2015)) also used it as baseline. Since our work is built upon those two papers, we decided to keep KLFA as baseline to keep the fidelity and replicability of the experiments.

The MOEAs’ initial population has a fixed size, depending on the algorithm. Comparing the initial population size between MOEAs and the state-of-art tool KLFA, the latter uses all the initial traces available for the generation of the model, whereas the former uses a limited set of individuals stochastically chosen. For NSGA-II, the population size and the offspring size are set respectively to 1,000 and 250 individuals as done in previous work (Tonella et al. 2012). For NSGA-III we follow the settings of the paper that proposes it (Deb and Jain 2014), by having the population size set to 91, almost equal to the number of reference points, which in our case (for 3 objectives and 12 partitions) is equal to 91. Similarly to NSGA-III, for MOEA/D we follow the paper that proposes it (Zhang and Li 2007), thus the population size was set to 300 and we let all the sub-problems to evolve equally by setting the number of offspring equal to the population size. Moreover, in MOEA/D the number of neighbours for each sub-problem is set to 20, and the Tchebycheff approach is used as decomposition technique because it is more appropriate for a discrete problem. Lastly, we perform objective normalization (Zhang and Li 2007) in order to deal with disparately scaled objectives.

These differences in population sizes are due to the different peculiarities of each algorithm. Whilst NSGA-II population size was kept the same as in previous work (Tonella et al. 2012), after a preliminary set of experiments, we discovered that 1,000 solutions would not be feasible for NSGA-III and MOEA/D due to their greater running time cost which estimates to twice or thrice more. With the recommended values for the latter two, we could successfully execute them in feasible time and with acceptable results.

In order to achieve a fair comparison, given the different starting population sizes, for all three algorithms, the termination condition is set to 26,000 maximum fitness evaluations or 18 hours, whichever comes first. We added a hard-limit on the execution time due to the elevated cost of the Hopcroft minimisation algorithm used during the the fitness function evaluation. In cases where the evaluated automata were considerably larger than the others, the evaluation of such automata would be too costly to the algorithm. Furthermore, during the evolutionary process and at the end of the generation, the solutions with OA and size values greater than 500 were removed, given that models with such size and infeasible traces are not useful in practice for the testers.

The crossover and mutation probabilities are set to 100% and 55% respectively based on previous work (Zhang et al. 2015). Indeed, the mutation rate is rather large when compared to conventional evolutionary algorithms (Zitzler et al. 2004). However, unlike regular mutation rates that are rolled for each gene in a chromosome to check whether the gene will be mutated, ours is rolled only once to decide whether the entire chromosome will face mutation or not. While regular mutations aim at mutating each child at least once, ours focuses on mutating only half of the children due to the more complex nature of the mutation operators. Furthermore, because a mutation may not be applied after all (recall the constraint of Random k-tails Merge in Section 3.5.4), a high mutation probability is required.

All experiments are performed on a machine with 32 GB of 3,200MHz RAM and an AMD Ryzen Threadripper 2950X 16-Core/32-Threads CPU. The execution time of all experiments were computed from the beginning of the search, until the very end, including deletion of dominated solutions.

### Experimental analysis

To address the stochastic nature of the evolutionary algorithms, we perform 30 independent runs, as suggested by several authors who give advise on SBSE experiments procedures (Arcuri and Briand 2014; Harman et al. 2010). After running all 30 runs for all algorithms on a dataset, we compute two performance indicators: HV and IGD. The two indicators are used respectively to evaluate the covered area of the objective space and the distance of a set of solutions from the reference pareto front. We use these indicators because they are well-known and widely used in literature for multi-objective problems (Riquelme et al. 2015; Barros 2012; Ishibuchi et al. 2006; Sarro et al. 2017; Guizzo et al. 2020; Tawosi et al. 2021). Secondly, because they fit our needs of properly comparing the algorithms solutions in terms of convergence and distribution of solutions in the objective space. Lastly these indicators are easily available and ready-to-use in most MOEAs frameworks.

In order to assess statistical significance and magnitudes of differences between algorithms, we have used Kruskal-Wallis Ranked Sum (Arcuri and Briand 2014) for the *p*-value testing and Vargha-Delaney $\hat{A}_{12}$ for the effect size (Arcuri and Briand 2014). We chose these statistical tests because they are non-parametric (do not assume normal distribution of the data) and can be easily applied to the MOEAs’ results. Moreover, Kruskal-Wallis provides one *p-value* for a group of two or more algorithms, unveiling whether there is a difference between the algorithms, and making it suitable for our purpose.

For both performance indicators, we normalise the objectives by computing the upper and lower bounds for each subject program by taking the lower and the higher objective values from all fitness vectors from the 30 runs of the three algorithms. For IGD we also collect the reference pareto front as the union of all models from all pareto fronts taken from the 30 runs of all three algorithms, as done in previous work (Sarro et al. 2016, 2017; Tawosi et al. 2021; Guizzo et al. 2020).

## Results

In this section we show the results and answer RQs 1–3.

### RQ1: comparison with KLFA

Differently from the MOEAs, KLFA only generates one solution (model) for each program, thus using quality indicators (as done in the next section) to compare their results would be unfair. With this in mind, we report objective results for the comparison between the algorithms and KLFA in Table 3. It is important to note that in this section we only compare the 3 objectives formulation UA-Size-OA for all MOEAs. The comparison between MOEAs themselves is presented in Section 5.2, and Section 5.3 presents the comparison between the 2 and 3 objectives formulations.

**Table 3:** **RQ1 & RQ2 & RQ3 –** Objectives and performance metrics results for NSGA-III, MOEA/D, and NSGA-II$_{ 3obj }$ with the UA-Size-OA formulation, NSGA-II$_{ 2obj }$ with the UA-OA formulation, and KLFA. The best values are highlighted in bold

		Mean (min, max)					
Program	Algorithm	UA	Size	OA	N. of Edges	N. of Solutions	Running Time
Kate	NSGA-II$_{ 2obj }$	233.7 (213, 254)	441.9 (408, 468)	**6.2** (0, 102)	604	3	4h 16m 15s
	NSGA-II$_{ 3obj }$	316.5 (179, 400)	**133.6** (1, 500)	98.4 (0, 500)	**234** (1, 917)	**379** (274, 453)	1h 40m 48s
	NSGA-III$_{ 3obj }$	333 (246, 400)	165.6 (1, 500)	128.4 (0, 500)	284 (1, 1053)	32 (18, 68)	3h 55m 35s
	MOEA/D$_{ 3obj }$	318.2 (205, 401)	175.9 (1, 500)	99.9 (0, 500)	290 (1, 901)	140 (72, 189)	5h 19m 16s
	KLFA	**0**	475	6000000	18802	1	**41m 46s**
Vibe	NSGA-II$_{ 2obj }$	301.4 (290, 312)	452.8 (403, 494)	**4.3** (0, 41)	564	3	1h 35m 11s
	NSGA-II$_{ 3obj }$	344.5 (242, 425)	174.1 (1, 500)	40.6 (0, 500)	**256** (1, 767)	**470** (392, 576)	1h 19m 37s
	NSGA-III$_{ 3obj }$	362.4 (289, 425)	186.7 (1, 499)	118.3 (0, 499)	274 (1, 854)	52 (20, 89)	2h 35m 46s
	MOEA/D$_{ 3obj }$	348 (272, 425)	208.4 (1, 500)	96.4 (0, 500)	310 (1, 786)	132 (101, 157)	4h 06m 31s
	KLFA	**0**	**88**	6000000	4989	1	**2m 24s**
Krita	NSGA-II$_{ 2obj }$	781.9 (755, 812)	444.9 (401, 480)	**15.6** (0, 422)	603	4	3h 29m 08s
	NSGA-II$_{ 3obj }$	859.4 (711, 958)	**140.2** (1, 500)	134.6 (0, 500)	**300** (1, 1155)	**473** (378, 559)	3h 44m 38s
	NSGA-III$_{ 3obj }$	889.3 (803, 958)	161.8 (1, 498)	138.2 (0, 500)	317 (1, 1548)	21 (16, 43)	8h 49m 33s
	MOEA/D$_{ 3obj }$	867.5 (748, 958)	180.5 (1, 500)	121.8 (0, 499)	330 (1, 1063)	131 (45, 187)	7h 09m 41s
	KLFA	**0**	301	15000000	34420	1	**2h 21m 23s**
LO Writer	NSGA-II$_{ 2obj }$	869.5 (848, 893)	450.5 (396, 485)	**12.4** (0, 245)	594	4	**2h 52m 46s**
	NSGA-II$_{ 3obj }$	925.3 (781, 1039)	164.9 (1, 499)	84.8 (0, 500)	288 (1, 1035)	**469** (358, 598)	3h 02m 41s
	NSGA-III$_{ 3obj }$	968.9 (869, 1040)	**140.5** (1, 500)	140.4 (0, 500)	**265**	24	8h 09m 04s
	MOEA/D$_{ 3obj }$	946.2 (833, 1040)	171.2 (1, 500)	104.6 (0, 500)	292	136	7h 23m 14s
	KLFA	**0**	609	15000000	61236	1	11h 29m 35s
Firefox OS	NSGA-II$_{ 2obj }$	918.9 (899, 939)	457.6 (407, 487)	**16.6** (0, 217)	588	4	3h 45m 37s
	NSGA-II$_{ 3obj }$	991.1 (849, 1082)	**141.9** (1, 500)	113.9 (0, 500)	**221** (1, 861)	**367** (268, 488)	4h 49m 36s
	NSGA-III$_{ 3obj }$	1007.3 (906, 1082)	160.5 (1, 496)	104.4 (0, 497)	239 (1, 844)	22 (15, 39)	11h 13m 54s
	MOEA/D$_{ 3obj }$	998.3 (870, 1082)	165.7 (1, 500)	76.4 (0, 500)	253 (1, 849)	159 (53, 190)	6h 31m 54s
	KLFA	**0**	279	15000000	31785	1	**2h 15m 20s**
Firefox And.	NSGA-II$_{ 2obj }$	883.1 (868, 902)	451.7 (401, 487)	**8.8** (0, 208)	593	4	2h 50m 53s
	NSGA-II$_{ 3obj }$	941.9 (799, 1054)	177.4 (1, 500)	61.9 (0, 500)	285 (1, 1028)	**460** (364, 587)	**2h 37m 24s**
	NSGA-III$_{ 3obj }$	987.8 (890, 1054)	**166.2** (1, 497)	116.5 (0, 500)	**278**	26	9h 42m 44s
	MOEA/D$_{ 3obj }$	957 (847, 1054)	205.1 (1, 500)	133.5 (0, 499)	332	126	7h 41m 17s
	KLFA	**0**	401	15000000	46246	1	4h 42m 03s
SeaMonkey	NSGA-II$_{ 2obj }$	1340.8 (1311, 1372)	446.4 (368, 477)	**18.0** (0, 455)	654	4	**4h 37m 27s**
	NSGA-II$_{ 3obj }$	1465.8 (1236, 1598)	**128.7** (1, 500)	103.8 (0, 500)	**247** (1, 1086)	**411** (332, 514)	7h 21m 46s
	NSGA-III$_{ 3obj }$	1513.2 (1370, 1598)	131.3 (1, 496)	142.8 (0, 496)	250 (1, 1001)	20 (12, 29)	15h 25m 14s
	MOEA/D$_{ 3obj }$	1473.6 (1327, 1598)	178.8 (1, 500)	104.6 (0, 499)	323 (1, 939)	176 (139, 208)	9h 13m 29s
	KLFA	**0**	945	15000000	96760	1	17h 46m 03s
Thunderbird	NSGA-II$_{ 2obj }$	1464.1 (1442, 1486)	455.1 (378, 489)	**26.6** (0, 480)	610	4	**3h 01m 00s**
	NSGA-II$_{ 3obj }$	1540.8 (1377, 1652)	**147.4** (1, 500)	99 (0, 500)	269 (1, 1187)	**432** (325, 515)	5h 58m 15s
	NSGA-III$_{ 3obj }$	1575.4 (1471, 1652)	157.5 (1, 500)	136.1 (0, 500)	**266** (1, 1304)	24 (17, 35)	15h 03m 44s
	MOEA/D$_{ 3obj }$	1556.2 (1415, 1654)	167.9 (1, 500)	98.8 (0, 497)	287 (1, 915)	167 (46, 215)	8h 50m 02s
	KLFA	**0**	391	15000000	56258	1	20h 06m 24s
Calendar	NSGA-II$_{ 2obj }$	2365.7 (2330, 2392)	457.2 (415, 484)	**27.5** (0, 362)	619	5	**3h 15m 08s**
	NSGA-II$_{ 3obj }$	2449.7 (2262, 2574)	**136.3** (1, 500)	108.8 (0, 500)	**269** (1, 1142)	**443** (331, 533)	8h 35m 44s
	NSGA-III$_{ 3obj }$	2489.9 (2367, 2573)	152.5 (1, 499)	142.7 (0, 499)	277 (1, 1245)	19 (15, 28)	17h 44m 25s
	MOEA/D$_{ 3obj }$	2467.4 (2332, 2576)	175.3 (1, 500)	105.4 (0, 500)	311 (1, 998)	167 (68, 194)	10h 06m 42s
	KLFA	**0**	209	15000000	38677	1	11h 32m 41s
BIRT	NSGA-II$_{ 2obj }$	4066.8 (4026, 4103)	448.7 (380, 479)	**24.4** (0, 479)	599	4	3h 18m 27s
	NSGA-II$_{ 3obj }$	4163.9 (3960, 4355)	139.2 (1, 500)	111.3 (0, 500)	251 (1, 1003)	**343** (277, 430)	12h 53m 10s
	NSGA-III$_{ 3obj }$	4225.3 (4096, 4360)	112.8 (1, 499)	148.1 (0, 498)	**212** (1, 852)	18 (14, 25)	18h 10m 41s
	MOEA/D$_{ 3obj }$	4163.1 (4012, 4361)	185.1 (1, 500)	116.1 (0, 498)	330 (1, 992)	147 (47, 193)	10h 57m 03s
	KLFA	**0**	**65**	15000000	6316	1	**2h 49m 11s**

The search-based algorithms have lower over-approximation values than KLFA, whereas the latter has always 0 under-approximation given by the technique’s behaviour which considers all the initial traces given as input. This is in line with the previous findings of Tonella et al. (2012) on a different set of subjects (2 software projects) with the same three objective functions.

The multi-objective algorithms are able to generate very small models compared to those of KLFA on average. In the extreme case, models only contain a single state. Although this kind of models show a low over-approximation, they are so small that they are not useful in practice, because they do not allow to test a big part of an application’s execution traces.

We now focus on the bug revealing ability of the MOEAs when compared to KLFA. In this specific scenario, the traces generated by the models are used to help the tester find new bugs, i.e., the tester needs to evaluate the traces and create test cases that can exercise the behaviours represented by such traces. This analysis is done in a cross-validation fashion over the test set (20% of the data) with the models generated using the training dataset (80% of the data), as explained in Section 4. We are interested in maximising the number of new bugs that can be revealed with the minimum number of traces possible (analysing traces and creating test cases is a costly task). We set the maximum length of traces during our data analysis to 6 because it is the average length of bug traces in all datasets, and because it is the best trade-off between results accuracy and computational effort.^5^

Table 4 shows this comparison. The third column contains the average number of traces up to length 6 contained inside all the solutions of a Pareto front. The fourth column shows the average number of bugs detected from all solutions of a Pareto front. The fifth column reports the total number of bugs found by all models of all Pareto fronts. The last column shows the ratio between average traces and average bugs detected, giving us the actual fault-revealing value.

**Table 4:** **RQ1 & RQ2 & RQ3 –** Results of the fault-revealing ability of the models inferred by NSGA-III, MOEA/D, and NSGA-II$_{ 3obj }$ with the UA-Size-OA formulation, and NSGA-II$_{ 2obj }$ with the UA-OA formulation. Column *A. #T* shows the average number of traces generated (up to size 6) per Pareto front, *A. #B* the average number of bugs revealed per Pareto front, *T. #B* the total number of distinct bugs revealed, and *A. #T/#B* the average number of traces needed to reveal each bug per Pareto front. The best values are highlighted in bold

Program	Algorithm	A. #T	A. #B	T. #B	A. #T/#B
Kate	NSGA-II$_{ 2obj }$	**286**	5.6	7	**51**
	NSGA-II$_{ 3obj }$	77 772	**7.4**	**14**	10 510
	NSGA-III$_{ 3obj }$	9 421	4.6	9	2 048
	MOEA/D$_{ 3obj }$	30 566	6.3	13	4 852
Vibe	NSGA-II$_{ 2obj }$	**213**	0.6	1	**355**
	NSGA-II$_{ 3obj }$	61 490	**1.0**	**3**	61 490
	NSGA-III$_{ 3obj }$	18 013	0.8	2	22 516
	MOEA/D$_{ 3obj }$	36 874	**1.0**	**3**	36 874
Krita	NSGA-II$_{ 2obj }$	**413**	10.6	15	**39**
	NSGA-II$_{ 3obj }$	146 307	**22.4**	**42**	6 532
	NSGA-III$_{ 3obj }$	5 977	10.9	31	548
	MOEA/D$_{ 3obj }$	38 965	14.2	37	2 744
LO Writer	NSGA-II$_{ 2obj }$	**325**	14.7	22	**22**
	NSGA-II$_{ 3obj }$	112 588	**25.3**	**43**	4 450
	NSGA-III$_{ 3obj }$	7 494	15.1	31	496
	MOEA/D$_{ 3obj }$	33 852	17.8	38	1 902
Firefox OS	NSGA-II$_{ 2obj }$	**343**	3.8	10	**90**
	NSGA-II$_{ 3obj }$	79 204	**4.8**	12	16 501
	NSGA-III$_{ 3obj }$	4 310	1.7	11	2 535
	MOEA/D$_{ 3obj }$	28 802	4.4	**13**	6 546
Firefox And.	NSGA-II$_{ 2obj }$	**318**	5.7	11	**56**
	NSGA-II$_{ 3obj }$	93 353	**10.7**	**32**	8 725
	NSGA-III$_{ 3obj }$	7 847	4.6	17	1 706
	MOEA/D$_{ 3obj }$	44 056	7.8	22	5 648
SeaMonkey	NSGA-II$_{ 2obj }$	**426**	12.0	23	**36**
	NSGA-II$_{ 3obj }$	97 405	**19.9**	**44**	4 895
	NSGA-III$_{ 3obj }$	4 747	10.1	31	470
	MOEA/D$_{ 3obj }$	31 184	15.4	34	2 025
Thunderbird	NSGA-II$_{ 2obj }$	**542**	9.4	20	**58**
	NSGA-II$_{ 3obj }$	74 994	**17.6**	**39**	4 261
	NSGA-III$_{ 3obj }$	4 850	7.7	30	630
	MOEA/D$_{ 3obj }$	29 596	12.5	33	2 368
Calendar	NSGA-II$_{ 2obj }$	**484**	11.2	31	**43**
	NSGA-II$_{ 3obj }$	97 553	**22.3**	**49**	4 375
	NSGA-III$_{ 3obj }$	4 765	9.8	36	486
	MOEA/D$_{ 3obj }$	29 598	15.5	44	1 910
BIRT	NSGA-II$_{ 2obj }$	**458**	26.9	69	**17**
	NSGA-II$_{ 3obj }$	90 656	**46.1**	97	1 967
	NSGA-III$_{ 3obj }$	6 391	24.5	69	261
	MOEA/D$_{ 3obj }$	32 615	34.8	**100**	937

In line with previous studies (Zhang et al. 2015; Tonella et al. 2012), we also observe that KLFA generates an impractical number of test traces, between 6.3 and 11,459 times more tests traces (up to length 6) on average with respect to the worst performing search-based approach depending on the software tested. This makes latter models preferable, even if they may find fewer bugs, since the cost involved in checking the results of test sequence requires human effort and it becomes outright infeasible with KLFA. Moreover, we could not generate traces for some programs due to the infeasible amount of computational resources needed for this task. For this reason, we have omitted the fault-revealing results of KLFA from Table 4.

Overall, we can state that the MOEAs generate more preferable models in practice because KLFA models have a too high over-approximation and this reflects on the number of edges and on the overall model complexity. In general, the size of a model reflects on the over-approximation, however, in the KLFA case, the models have a low number of states and a high over-approximation due to the numerous loops (or self-transitions) inside the model. Therefore, the KLFA models tend to over-approximate rather than under-approximate an application’s behaviour, resulting in very specific but too complex models for the targeted problem (Tonella et al. 2012).
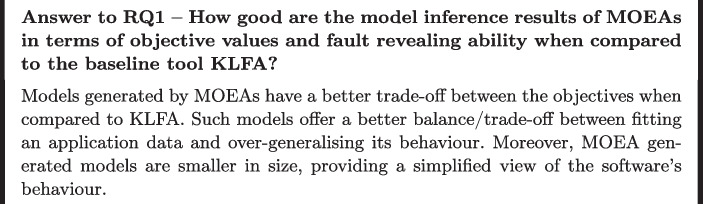


### RQ2: different MOEAs

In this RQ we are interested in comparing the results obtained by NSGA-II, NSGA-III, and MOEA/D. To assess the multi-objective quality of the resulting Pareto fronts, we compute the HV and IGD indicator (Zitzler et al. 2004) values for each of the 30 independent runs, and report the averages and standard deviations in Table 5. Columns five and nine show the *p*-value obtained by the the Kruskal-Wallis Ranked Sum test based on multi-comparison for HV and IGD results respectively. Values in bold represent the best results.

**Table 5:** **RQ2 –** HV and IGD results and *p*-values for NSGA-II, NSGA-III and MOEA/D. The greater the HV, the better. The lower the IGD, the better

Program	NSGA-II	NSGA-III	MOEA/D	*p-value*
	Hypervolume (HV)			
Kate	**0.60 (0.03)**	0.35 (0.04)	0.46 (0.02)	< 2.2e-16
Vibe	**0.60 (0.01)**	0.40 (0.03)	0.46 (0.02)	< 2.2e-16
Krita	**0.60 (0.02)**	0.31 (0.03)	0.43 (0.03)	< 2.2e-16
LO Writer	**0.60 (0.02)**	0.32 (0.04)	0.44 (0.03)	< 2.2e-16
Firefox OS	**0.58 (0.02)**	0.36 (0.04)	0.47 (0.02)	< 2.2e-16
Firefox And.	**0.59 (0.01)**	0.29 (0.05)	0.41 (0.02)	< 2.2e-16
SeaMonkey	**0.58 (0.02)**	0.27 (0.04)	0.43 (0.02)	< 2.2e-16
Thunderbird	**0.60 (0.02)**	0.32 (0.02)	0.45 (0.03)	< 2.2e-16
Calendar	**0.61 (0.01)**	0.30 (0.04)	0.43 (0.03)	< 2.2e-16
BIRT	**0.67 (0.02)**	0.38 (0.03)	0.54 (0.04)	< 2.2e-16
	IGD			
Kate	**0.05 (0.003)**	0.16 (0.02)	0.10 (0.01)	< 2.2e-16
Vibe	**0.05 (0.006)**	0.12 (0.02)	0.09 (0.01)	< 2.2e-16
Krita	**0.05 (0.005)**	0.19 (0.02)	0.11 (0.02)	< 2.2e-16
LO Writer	**0.05 (0.005)**	0.20 (0.04)	0.11 (0.02)	< 2.2e-16
Firefox OS	**0.05 (0.006)**	0.16 (0.02)	0.08 (0.02)	< 2.2e-16
Firefox And.	**0.05 (0.005)**	0.22 (0.04)	0.41 (0.02)	< 2.2e-16
SeaMonkey	**0.05 (0.004)**	0.27 (0.03)	0.43 (0.01)	< 2.2e-16
Thunderbird	**0.05 (0.004)**	0.18 (0.02)	0.09 (0.02)	< 2.2e-16
Calendar	**0.05 (0.003)**	0.20 (0.03)	0.10 (0.02)	< 2.2e-16
BIRT	**0.05 (0.004)**	0.21 (0.03)	0.09 (0.02)	< 2.2e-16

As a sanity check, we compared the results of all algorithms against a Random Search algorithm with the same search budget. For all comparisons in all cases, the results of the Random Search algorithm were worse than NSGA-II, NSGA-III, and MOEA/D, yielding *p*-values lower than 0.05 and always large effect sizes in favour of the other algorithms. Therefore, we can state that the algorithms used in this paper are not obtaining their results due to pure chance, but rather are actively searching for solutions in the multi-objective search space. For sake of space, we do report herein the full results of this comparison in our replication package.^6^

The best algorithm in all the scenarios and data we tested^7^ is NSGA-II, which achieves the highest HV and the lowest IGD mean values on all training programs with statistical significance. The HV results reveal how NSGA-II is able to produce more diverse and dominating solutions in the objective space. This is really important because the algorithm offers more choices for testers who have to select the best model among all the non-dominated solutions which have different objectives trade-offs, based on the application needs. On the other hand, the IGD results show that NSGA-II is capable of generating solutions which represent the best Pareto set approximation for the inference problem at hand.

In order to assess the magnitude of HV and IGD values difference between the MOEAs, we compute the Vargha-Delaney $\hat{A}_{12}$ effect size. Table 6 shows the effect size results.

**Table 6:** **RQ2 –** HV and IGD effect size results for NSGA-II, NSGA-III and MOEA/D. Greater HV effect sizes and lower IGD effect sizes are better for Algorithm X. Effect sizes of 0.5 indicate no difference

Program	Alg. X	Alg. Y	HV	IGD
Kate	NSGA-II	NSGA-III	1	0
	NSGA-II	MOEA/D	1	0
	NSGA-III	MOEA/D	0.012	0.961
Vibe	NSGA-II	NSGA-III	1	0
	NSGA-II	MOEA/D	1	0
	NSGA-III	MOEA/D	0.056	0.911
Krita	NSGA-II	NSGA-III	1	0
	NSGA-II	MOEA/D	1	0
	NSGA-III	MOEA/D	0.002	0.992
LO Writer	NSGA-II	NSGA-III	1	0
	NSGA-II	MOEA/D	1	0
	NSGA-III	MOEA/D	0.017	0.992
Firefox OS	NSGA-II	NSGA-III	1	0
	NSGA-II	MOEA/D	1	0.003
	NSGA-III	MOEA/D	0.008	0.992
Firefox And.	NSGA-II	NSGA-III	1	0
	NSGA-II	MOEA/D	1	0
	NSGA-III	MOEA/D	0.038	0.962
SeaMonkey	NSGA-II	NSGA-III	1	0
	NSGA-II	MOEA/D	1	0
	NSGA-III	MOEA/D	0.006	1
Thunderbird	NSGA-II	NSGA-III	1	0
	NSGA-II	MOEA/D	1	0
	NSGA-III	MOEA/D	0.025	0.861
Calendar	NSGA-II	NSGA-III	1	0
	NSGA-II	MOEA/D	1	0
	NSGA-III	MOEA/D	0.011	0.999
BIRT	NSGA-II	NSGA-III	1	0
	NSGA-II	MOEA/D	0.991	0
	NSGA-III	MOEA/D	0.003	0.998

For all programs, NSGA-II has 100% probability of having higher HV values than NSGA-III and MOEA/D. Only the biggest dataset BIRT shows an effect size value not equal to one for the comparison between NSGA-II and MOEA/D, however the probability is still near to 100% (99%). Furthermore, NSGA-II has 100% probability of having lower IGD values (the lower the better) than the other two algorithms.

The effect size analysis is a clear evidence of how NSGA-II solutions are better in all cases, both for solutions diversity and optimality (distance from the reference Pareto front). The magnitude of the HV and IGD difference is always largely in favour of NSGA-II, making it preferable over the other two evolutionary algorithms.

We further report the objective results for the comparison between the algorithms in Table 3. Note that NSGA-III and MOEA/D use the 3 objectives formulation, thus in this section we compare the results of NSGA-II with the 3 objectives formulation.

While the minimum and maximum values of model size and over-approximation are almost identical for the three MOEAs, the average values show a consistent difference. For 70% of the datasets, NSGA-II$_{ 3obj }$ has lower size values, reflecting the overall model complexity on the number of edges as well where NSGA-II produces better values for 100% of the training sets. For six out of ten projects it has lower over-approximation values, meaning that NSGA-II models can better fit a specific problem without over generalising as often. Lastly, the under-approximation for NSGA-II is better for 80% of the projects, resulting in models which are unable to recognize fewer application bugs.

What is evident from the results is that NSGA-II has a higher number of solutions for each Pareto front (depending on the initial population size) and consistently lower average objectives values compared to those of NSGA-III and MOEA/D, confirming the solutions generated by the non-dominated sorting strategy the best for the model-inference problem.

We further analyse the results of each MOEA in terms of fault revealing capabilities, as shown in Table 4. NSGA-II has the highest number of total bugs found for 80% of the programs. An exception is the BIRT dataset, in this case MOEA/D has more bugs found, with a significantly lower number of tests per bug revealed. Although NSGA-III has the lowest test per bug revealed ratio, the number of bugs found is always lower than the others. Overall, MOEA/D seems to show the best trade-off between bugs found and average tests per bug because it can reveal more bugs than NSGA-III with a lower number of traces than NSGA-II, while keeping the average test per bug revealed smaller.
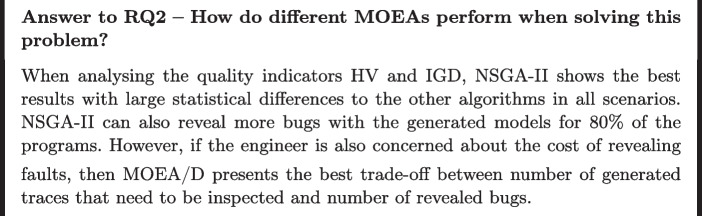


### RQ3: 2 objectives vs. 3 objectives

RQ3 focuses on assessing the effectiveness of a three-objectives formulation compared to a two-objectives one. Since RQ2 revealed that NSGA-II is the best algorithm according to performance indicators, we perform the objectives combination comparisons using this algorithm. We compare the three-objectives formulation UA-Size-OA with the two-objectives formulations UA-Size, OA-Size, and UA-OA.

After some preliminary tests, we discovered that the combinations UA-Size and OA-Size are not feasible for the model inference problem. Taking the example of the smallest dataset Kate, in the OA-Size formulation the replacement operator allows individuals with very few states (the average size of a model is 1.84) to survive. Focusing only on those two objectives leads to infeasible models in practice because of their small size and lack of generalisation to real bug traces. On the other hand, in the UA-Size formulation, even though the model size is kept relatively small (150.02 states on average), it leads to infeasible models on the opposite front. The final solutions are more complex and they have an higher over-approximation than the UA-Size-OA formulation, making them practically infeasible to analyze because of the high number of traces (tens of millions). Both combinations also show a lack of solution diversity (the average number of solutions is 14), producing fewer unique and non-dominated solutions compared to the three objectives combination.

Given that the previous two objectives formulations are infeasible for two opposite reasons (either too simple or too complex models), in the remaining we focus on the comparison of NSGA-II with UA-Size-OA (NSGA-II$_{ 3obj }$) and NSGA-II with UA-OA (NSGA-II$_{ 2obj }$). Table 3 shows the objectives values, together with the average number of edges and the average number of solutions in a Pareto front. We compute the size values for the two objectives combination afterwards as they were not included in the evolutionary process.

The Pareto fronts of the 30 independent runs of NSGA-II$_{ 2obj }$ contain very few non-dominated and unique solutions on average, as opposed to NSGA-II$_{ 3obj }$, which provides hundreds of solutions. This is an evidence that the algorithm tends to early converge towards a local optimum, exploring just a few locally good solutions. Moreover, when the size objective is removed from the search, the models grow significantly in size (from twice to thrice as many edges).

We now investigate the fault-revealing ability of the two formulations. Table 4 shows the fault revealing ability analysis results.

NSGA-II$_{ 3obj }$ produces models with more traces, bugs, and test per bug revealed. The fault revealing ability (average and total number of bugs revealed) of NSGA-II$_{ 2obj }$ is not as high as the three-objectives formulation. NSGA-II$_{ 3obj }$ is consistently better on all datasets in terms of bugs detected, so it can achieve more powerful models.

However, due to the great number of traces that can be produced by NSGA-II$_{ 3obj }$, the average number of traces per bug revealed is several orders of magnitude higher than NSGA-II$_{ 2obj }$. Therefore, in order to reveal a new bug, the engineer has to evaluate 100–1,000 times more traces on average, either manually or automatically using test case generation. This can increase the overall cost fo the testing activity, but not necessarily make it infeasible.

Another interesting observation is the fact that NSGA-II$_{ 2obj }$ produces bigger models, but ultimately its set of models produce fewer traces than the set of smaller models of NSGA-II$_{ 3obj }$. Not using the size objective can also explain the low over-approximation of the models. However, the diversity of solutions introduced by NSGA-II$_{ 3obj }$ allows the generation of more unique traces, and consequently reveal more bugs. Hence, for this problem, having more succinct models in the Pareto front is more beneficial to the overall fault revealing ability of the testing task than having fewer and more complex models.

Table 7 presents the HV and IGD results, followed by the *p*-values and Vargha-Delaney Â_12_ effect size values for both the indicators. Since, NSGA-II$_{ 2obj }$ did not optimise for the size objective, in this particular analysis, we computed the quality indicators taking into account only the UA and OA objectives. That way, we can guarantee a more fair comparison between the formulations.

**Table 7:** **RQ3 –** HV and IGD results, *p*-values, and effect sizes (ES) for NSGA-II$_{ 3obj }$ and NSGA-II$_{ 2obj }$. The greater the HV, the better. The lower the IGD, the better. Greater HV effect sizes and lower IGD effect sizes are better for NSGA-II$_{ 3obj }$. Effect sizes of 0.5 indicate no difference

Program	NSGA-II$_{ 3obj }$	NSGA-II$_{ 2obj }$	*p-value*	ES
	Hypervolume (HV)			
Kate	**0.86 (0.07)**	0.78 (0.02)	< 6.5e-06	0.84
Vibe	**0.91 (0.02)**	0.69 (0.02)	< 2.5e-11	1
Krita	**0.86 (0.03)**	0.75 (0.03)	< 6.9e-11	0.99
LO Writer	**0.88 (0.04)**	0.68 (0.02)	< 2.6e-11	1
Firefox OS	**0.89 (0.04)**	0.73 (0.02)	< 2.7e-11	1
Firefox And.	**0.91 (0.03)**	0.70 (0.02)	< 2.6e-11	1
SeaMonkey	**0.82 (0.06)**	0.74 (0.02)	< 2.1e-07	0.89
Thunderbird	**0.90 (0.03)**	0.72 (0.02)	< 2.6e-11	1
Calendar	**0.89 (0.04)**	0.70 (0.03)	< 2.7e-11	1
BIRT	**0.88 (0.04)**	0.76 (0.02)	< 4.9e-11	0.99
	IGD			
Kate	**0.02 (0.006)**	0.47 (0.03)	< 9.6e-12	0
Vibe	**0.02 (0.003)**	0.32 (0.01)	< 3.3e-12	0
Krita	**0.02 (6.9e-18)**	0.47 (0.04)	< 1.1e-12	0
LO Writer	**0.02 (0.002)**	0.35 (0.03)	< 2.1e-12	0
Firefox OS	**0.02 (0.004)**	0.43 (0.04)	< 5.7e-12	0
Firefox And.	**0.02 (0.003)**	0.34 (0.02)	< 3.5e-12	0
SeaMonkey	**0.02 (0.004)**	0.44 (0.04)	< 5.8e-12	0
Thunderbird	**0.02 (0.002)**	0.38 (0.04)	< 2.1e-12	0
Calendar	**0.02 (6.9e-18)**	0.38 (0.04)	< 1.1e-12	0
BIRT	**0.02 (0.003)**	0.37 (0.04)	< 2.9e-12	0

NSGA-II$_{ 3obj }$ produces more diverse solutions with better objectives values compared to NSGA-II$_{ 2obj }$. In 100% of the datasets, NSGA-II$_{ 3obj }$ has higher values for HV and lower values for IGD. Moreover, the Kruskal-Wallis test showed statistical significance in all comparisons. NSGA-II$_{ 3obj }$ has almost 100% probability (effect size of 1 for HV and 0 for IGD) of having better values than NSGA-II$_{ 2obj }$. This translates into a large statistically significant difference between the formulations, for which the three objectives formulation always yields the best results.

Taking all the results of this subsection into consideration, we can state that the size objective is needed in the problem formulation as it can be seen as a controller for the number of vertices in a model (when we do not use the size objective, the sizes vary without control), but also as a diversity mechanism during the evolutionary process. Such an objective helps maintain smaller and succinct models, whilst also increasing the diversity during the search.

What is also evident from the results is that the size objective does not control for the number of possible traces, which is the parameter that we are interested in controlling to keep the models cheap to analyse. Hence, other factors determining the traces complexity can be investigated in future work, for example the number of transitions, which can be considered as a proxy for the interpretability of a model, can be used to guide the evolutionary search.
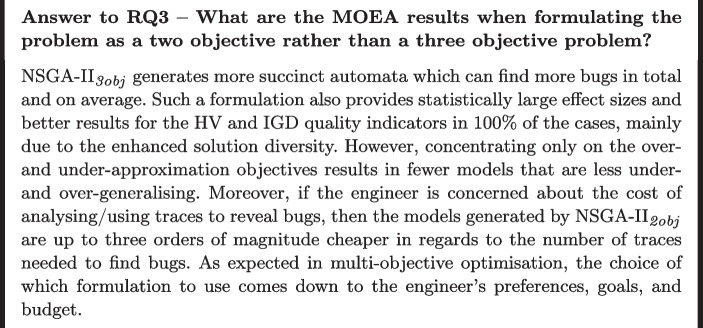


## Threats to validity

*Threats to Internal Validity*: We used pymoo (Blank 2022) as the framework for multi-objective optimisation. Each algorithm is implemented with its configurations and adjusted according to the available features in the framework. An incorrect implementation and/or configuration of algorithms can impact the final results of our evaluation. We carefully reviewed and configured each algorithm based on previous work recommendations (Zhang et al. 2015; Zhang and Li 2007; Deb et al. 2002; Deb and Jain 2014) before executing our experiments. Moreover, we also made sure to use the quality indicators with the recommended adjustments (Zitzler et al. 2004) (i.e., normalisation, and approximated reference fronts).

When looking for reproduction steps during the creation of the word dataset, it is possible that the reproduction steps are poorly described or structured by the users, thus leading to the creation of inaccurate models. If such model is used during training, then incorrect steps are created and the unobserved fitness will increase. If it is used during testing, the number of unrecognised traces will increase. In order to mitigate this threat, we search for and only include bug reports written in a well structured set of steps (itemised) and between specific keywords, and yet, we were able to mine at least 500 bugs for each program.

*Threats to Construct Validity*: In order to cater for the stochasticity of algorithms, we performed multiple independent runs and used statistical tests in our results, as recommended by Arcuri and Briand (2014). Furthermore, we evaluated our results using both Software Engineering focused measures (e.g., number of revealed bugs, size of models, and trace cost) and Multi-Objective Optimisation specific indicators (e.g., HV and IGD). With such approach, we intend to provide as much information as possible on our experimental results, consequently allowing the reader to evaluate such results in the best way they deem fit.

*Threats to External Validity*: The programs used in the experiments might not be representative of the true population of Software. In order to mitigate this threat, we collected programs used in previous work (Zhang et al. 2015) and added new programs of different sizes, number of bug reports, and states. However, we have not tested our approach against a newly created project with a limited number of bug reports, and thus we cannot generalise our results to this kind of project.

Similarly, the choice of MOEAs might not be representative of all MOEAs. The three MOEAs used in our work are well-know and common algorithms in the SBSE literature, mainly NSGA-II (Deb et al. 2002) which was also used in previous work (Zhang et al. 2015; Tonella et al. 2012). Moreover, we purposely selected different types of MOEAs (such as MOEA/D which is based in decomposition) to cater for the great variety of algorithms. We make our experimental data and code publicly available respectively at https://doi.org/10.5522/04/14736180 and https://github.com/SOLAR-group/ModelInference, to allow for reproduction and extension of our work.

DFA models have a limitation in regards to their applicability in practice. If the existing features of a software are not removed from the software or drastically changed in behaviour, the models work as expected and the correct traces are generated. However, new features would not be reproducible with the models because they do not have the correct states to capture new functionalities, and thus the number of unrecognised traces would increase. Similarly, if a feature is completely removed from the software, the number of unobserved traces would also increase, thus making the models not so effective. However, this is a general limitation of any model created from execution steps: they are susceptible to the natural evolution of the software and thus the engineer needs to maintain them updated when adding new features.

Moreover, we do not know how well our approach scale to bigger programs with a wider corpus of bug reports. For example, the manual creation of a synonym and typo dictionary (Section 3.4) may render our approach infeasible depending on the size of the software and list of bug reports. Future work should focus on assessing this scalability.

## Related work

Natural Language Processing (NLP) techniques applied to software testing allow for the extraction of useful knowledge from requirements and artifacts written in natural language. Such information allow us to automatically infer computational models, which can then be used to generate test cases, thus minimising both the high effort and potential errors caused by a manual approach.

Search-Based Model-Driven Engineering (SBMDE) investigates meta-heuristic search techniques to aid software engineers in a variety of Model-Driven Engineering tasks, such as automatically generating or optimizing both models and model transformations, automatically generating software test procedures by using models of system requirements and behaviour (Boussaïd et al. 2017). This has rapidly became a widely investigated topic in the literature given the tedious challenges posed by manual MDE, especially in the field of Model-Based Testing (MBT) (Boussaïd et al. 2017).

Due to the wide range of applications of both MBT and SBMDE, we focus our discussion on previous work at the intersection of NLP and search-based model inference testing. We refer the reader to the systematic mapping study of Garousi et al. (2020) for a comprehensive review of MBT using NLP-assisted techniques, to the survey by Boussaïd et al. (2017) for a comprehensive review of SBMDE approaches, to the systematic review of Saeed et al. (2016) for an extensive analysis on Search-Based Techniques for MBT, and to the survey of Harman et al. (2012) for a more general review of search-based techniques for all other areas of Software Engineering.

The current literature in search-based model testing aided by NLP, can be broadly divided in three categories: the one exploiting software requirements to infer model, the one using software execution traces, and the one using user bug reports. Work falling in each of these categories automatically generate test cases based on the inferred models exploiting a variety of approaches depending on the type of model, as detailed below.

### MBT using NLP and software requirements

Santiago Júnior and Vijaykumar (2012) present a model-based approach, dubbed SOLIMVA, which is able to generate test cases from natural language requirement deliverables. They use a tool which automatically translates requirements into Statechart models. Then, the GTSC tool is used to generate test cases. Their approach requires the definition of the application domain through a dictionary. Scenarios for system and acceptance testing are identified using combinatorial designs.

Carvalho et al. (2014) use a model-based testing approach that takes as input requirements formulated in a Controlled Natural Language. Requirements are syntactically analysed following a domain specific language used for describing system requirements and the semantics is described based on the Case Grammar theory. The semantics is then represented as transition relation, so inferring a model from which a solver can generate test cases.

Wang et al. (2020) presented an approach for generating acceptance test cases from requirements specifications in natural language. Their approach, UMTG, is based on use case specifications and a domain model for the system under test. The generation of test data relies on the use of NLP, adopted for automatically identifying test scenarios and generating formal constraints representing conditions that trigger the execution of the test scenarios. UMTG uses NLP to build Use Case Test Models (UCTMs) from Restricted Use Case Modeling (RUCM) specifications. The model captures the application control flow and enables the identification of the sequences of use case steps, and consequently the generation of executable test cases.

### Search-based MBT using NLP and execution traces

Tonella et al. (2012) investigated the problem of balancing over- and under-approximation in models inferred from execution traces. They achieved a balance of over- and under-approximation by applying two search-based algorithms: a multi-objective Genetic Algorithm (GA) and NSGA-II (Deb et al. 2002). They showed that “models generated by multi-objective algorithms are well-distributed across various levels of over- and under-approximation”. Moreover, they proved that the models produced by multi-objective algorithms violate fewer application constraints than the models generated by the KLFA tool (Tonella et al. 2012).

Subsequently, Tonella et al. (2014) proposed a method to automatically infer models from program execution traces, leveraging on the N-gram probabilistic language model. They apply this technique to obtain models that do not violate dependencies and constraints of the application. They adopt interpolated N-grams which are able to derive feasible test cases from a model. The interpolated N-grams overcome the limitation of N-grams statistics that, as N increases, becomes incomplete.

More recently, Shin et al. (2022) proposed PRINS, a component-based model inference approach using system logs. PRINS first generates models for each of the system’s components and then merges them together using information about their interactions. For generating the models, PRINS uses MINT (Walkinshaw et al. 2013b), an “off-the-shelf” tool that uses Search-based algorithms. Similarly, Liu et al. (2016) propose a component-based approach for inferring behavioural models by mining event logs. Other work also usually infer models from system logs, but the models are represented differently (Mariani et al. 2017) or are non-deterministic (Emam and Miller 2018).

The main difference between those works and ours is that we use a different approach to infer models from another source of information. We perform the extraction of steps and traces from a bug report system, apply many steps of the NLP pipeline to obtain a dictionary of “bug traces”, and only then we infer the models. Moreover, since bug reports are written by humans with no clear structure (as it happens in an execution log), we face different types of challenges.

### Search-based MBT using NLP and bug reports

Zhao et al. (2019) proposed ReCDroid, a tool to automatically reproduce crashes from bug reports. ReCDroid generates event sequences based on the bug reports, which can later be used by the engineers to guide their tests and reproduce the reported crashes. The focus of this tool is on reproducing GUI crashes. Differently from our work, ReCDroid only uses one objective and executes a restarting procedure whenever the set of GUI actions grows in size.

According to Saeed et al. (2016), using multi-objective search is one of the identified challenges in MBT. In their systematic literature review, only 14% of papers they identified used any kind of multi-objective optimisation. With that in mind and inspired by the work of Tonella et al. (2012, 2014), we envisaged that the use of natural language processing, coupled with the power of multi-objective optimisation, would allow us to automatically infer effective test models from user bug reports (Zhang et al. 2015).

Thus, Zhang et al. (2015) presented a proof of concept benchmarking this proposal against KLFA on a single software system (Kate) in the award winning SSBSE’2015 challenge paper. Herein, we fully present the idea, detail the process applied, and perform a large-scale empirical study in order to verify its applicability and effectiveness for a wider range of real software systems.

### Other related work

The work of Lucas and Reynolds (2005) presents an evolutionary algorithm for the automatic inference of DFA models. Although not related to Software Testing, their approach resembles ours in the evolutionary aspect. However, their representation and context significantly differ from ours.

First, as we describe in Section 3.5.1, our representation considers incomplete DFAs to represent impossible SUT behaviour, meaning that the transition matrix might not be complete. Moreover, our states do not have complex classes, i.e., only one state can be considered the final state and there is no output other than “accept” or “reject” for a given input. Finally, their algorithm works on the transition matrix using training and testing data, whereas ours focuses on inferring entire models (e.g., states, transitions, and transition matrix) from scratch using the entire set of traces.

The usage of more general approaches such as the one presented by Lucas and Reynolds (2005) would not be as suitable for the problem at hand because one of the three objectives is to reduce model over-approximation. In other words, we want to generate models that do not allow for the creation of impossible/unobservable action sequences in practice. Using an approach generating only complete DFAs would decrease the models’ under-approximation at the cost of increasing their over-approximation, as both measures are conflicting. In other words, the models would be more likely to accept a possible but unseen transition that would allow the discovery of new bugs. These models could then be pruned to become incomplete and to avoid impossible transitions (also unseen). However, impossible transitions and valid unseen transitions would likely be indistinguishable, because both are unseen in the training set. Therefore, instead of pruning states and transitions after a complete DFA is generated, previous work on automated test model generation has opted for the use of incomplete DFAs models by applying transformations that allow the generation of unseen traces (Zhang et al. 2015; Tonella et al. 2012). In this way, one can keep a low over-approximation while also being less likely to increase under-approximation. In this work, we adopted the same representation as in previous work (Zhang et al. 2015; Tonella et al. 2012), and the results of our experiments show that by using an evolutionary algorithm searching for both complete and incomplete DFA models (where no pruning is required) one can achieve the best trade-off between the two objectives of under-approximation and over-approximation.

What we observed with KLFA (and could likely happen with other more general approaches) is the generation of models that are so general that lead to too many unobservable traces, thus increasing model over-approximation which, in turn, makes their use infeasible in practice, i.e., an engineer cannot manually analyse that many traces in a reasonable time. Instead, based on related work and our own experience we found that allowing DFA models to be incomplete from the beginning of their inference, and constraining them to having only one initial state and one accepting state is more effective to reduce both the over- and under-approximation.

## Conclusion and future work

In this paper, we proposed and compared the use of three well-known MOEAs (NSGA-II, NSGA-III and MOEA/D) to automatically infer state-machine models. Our results showed that NSGA-II achieves significantly better results than all other approaches for all programs under investigation. Moreover, NSGA-II can detect a higher number of bugs for 90% of the programs studied with respect to the other MOEAs.

We also studied the differences in quality and models performance between guiding the MOEAs by using two objectives and three objectives. The combinations of under-approximation with size, and over-approximation with size, generated infeasible results. On the other hand, combining over-approximation with under-approximation generated feasible models that, although bigger in size, produced fewer traces in general and were cheaper when considering the number of traces needed to reveal bugs. However, using the three-objective formulation yielded better results, larger effect sizes, and statistically significant results in 100% of the cases. The size objective acts as a diversity factor, avoiding local optima, controlling the size of the models, making the results more diverse and nearer to the optimal Pareto set, and generating more balanced models for real-world scenarios.

As future work, the parameters of the MOEAs such as the number of reference points for NSGA-III or the population size can be automatically tuned to further improve the results. We reckon that different configurations would provide insightful results, thus we intend to explore these possibilities in future work. MOEA/D showed promising results when considering the objectives trade-off, hence a more thorough investigation of decomposition algorithms could yield better results (e.g., MOEA/DD and MOEA/D-CMA (Castro et al. 2017)). Furthermore, since the size objective was not able to maintain low model costs during evolution, future work can investigate whether other objectives such as number of transitions, can better capture the cost of the models, thus making them more practical. Another possible future work would be the incorporation of semantic analysis in the set of sentences and words, which could possible enhance the accuracy of the clustered steps used to build the models. User Interface exploration steps can also be considered in the set of available steps, thus creating a hybrid approach that leverages on both human written traces and automatically generated ones. Besides, future work can investigate and compare different clustering approaches to assess if the results obtained herein can be further improved. Last but not least, it would be also interesting to investigate the effectiveness of the models over time (i.e., model degradation) and across different projects to aid the use of the approach with newly-developed software lacking its own bug reports (cross-project models).

## Data Availability

The data generated and analysed during the current study is available in the UCL’s Figshare repository, https://doi.org/10.5522/04/14736180.

## Footnotes

1 Strictly speaking we originally presented this idea in a short paper for the Challenge Track of the Symposium on Search-Based Software Engineering (Zhang et al. 2015), which won the challenge. In this paper we provide the full proposal of the approach with a comprehensive description of its functionality, and a large-scale empirical evaluation.

2 https://www.nltk.org/

3 https://pypi.org/project/FAdo/

4 We set a maximum size of 4 due to the great computational cost of generating sets with bigger traces.

5 Not to be confused with the maximum length of 4 used during the optimisation process.

6 https://doi.org/10.5522/04/14736180

7 Note that we cannot generalise these results outside the scope of our work, as explained in Section 6.
